# Active components and molecular mechanisms of Sagacious Confucius’ Pillow Elixir to treat cognitive impairment based on systems pharmacology

**DOI:** 10.18632/aging.204912

**Published:** 2023-07-30

**Authors:** Zhitao Hou, Xinyu Yang, Ling Jiang, Liying Song, Yang Li, Dongdong Li, Yanning Che, Xiuling Zhang, Zhongren Sun, Hongcai Shang, Jing Chen

**Affiliations:** 1 College of Basic Medical and Sciences, Heilongjiang University of Chinese Medicine, Harbin, Heilongjiang 150040, China; 2 Key Laboratory of Chinese Internal Medicine of the Ministry of Education, Dongzhimen Hospital Affiliated with Beijing University of Chinese Medicine, Beijing 100700, China; 3 Department of Systems Pharmacology and Translational Therapeutics, Perelman School of Medicine, University of Pennsylvania, Philadelphia, PA 19104, USA; 4 Center for New Drug Research and Development, Harbin No. 4 Traditional Chinese Medicine Factory Co. Ltd., Harbin, Heilongjiang 150025, China; 5 Center for New Drug Research and Development, Heilongjiang Deshun Chang Chinese Herbal Medicine Co. Ltd., Harbin, Heilongjiang 150025, China; 6 Fangshan Hospital of Beijing University of Chinese Medicine, Beijing 102400, China; 7 Department of Clinical Medicine, Heilongjiang Nursing College, Harbin, Heilongjiang 150086, China

**Keywords:** active components, sagacious confucius’ pillow elixir, cognitive impairment, systems pharmacology, traditional Chinese medicine

## Abstract

Background: Sagacious Confucius’ Pillow Elixir (SCPE) is a common clinical prescription to treat cognitive impairment (CI) in East Asia.

Objective: To predict the active components of SCPE, identify the associated signaling pathway, and explore the molecular mechanism using systems pharmacology and an animal study.

Methods: Systems pharmacology and Python programming language-based molecular docking were used to select and analyze the active components and targets. Senescence-accelerated prone 8 mice were used as a CI model. The molecular mechanism was evaluated using the water maze test, neuropathological observation, cerebrospinal fluid microdialysis, and Western blotting.

Results: Thirty active components were revealed by screening relevant databases and performing topological analysis. Additionally, 376 differentially expressed genes for CI were identified. Pathway enrichment analysis, protein–protein interaction (PPI) network analysis and molecular docking indicated that SCPE played a crucial role in modulating the PI3K/Akt/mTOR signaling pathway, and 23 SCPE components interacted with it. In the CI model, SCPE improved cognitive function, increased the levels of the neurotransmitter 5-hydroxytryptamine (5-HT) and metabolite 5-hydroxyindole acetic acid (5-HIAA), ameliorated pathological damage and regulated the PI3K/AKT/mTOR signaling pathway. SCPE increased the LC3-II/LC3-I, p-PI3K p85/PI3K p85, p-AKT/AKT, and p-mTOR/mTOR protein expression ratios and inhibited P62 expression in the hippocampal tissue of the CI model.

Conclusion: Our study revealed that 23 active SCPE components improve CI by increasing the levels of the neurotransmitter 5-HT and metabolite 5-HIAA, suppressing pathological injury and regulating the PI3K/Akt/mTOR signaling pathway to improve cognitive function.

## INTRODUCTION

Cognitive impairment (CI) is a common cognitive disorder in older populations, affecting 5.7 million Americans currently. By 2050, it will affect 13.8 million Americans. Currently, the incidence of CI is gradually increasing; this increase is linked to population aging because senescence inhibits aging organelles from being cleared in a timely manner, accounting for the most important causes of CI [1]. CI usually has a rapid onset and progression related to aging, sleep deprivation, and diabetes, particularly among the elderly population [2]. The potential molecular mechanisms of CI, including the abnormal accumulation of β-amyloid (Aβ), phosphorylation of the Tau protein and theory of neuroinflammation, have been widely proven [3]. In recent years, studies have indicated that CI is closely related to autophagy and should be considered a self-metabolic disorder [4]. Currently, no effective treatments are available for CI, and most patients receive clinical protocols that merely provide symptomatic support [4, 5].

For the last thousand years, Oriental medicine has used a well-defined approach to treat CI [6, 7]. To date, Kaixin San [8], Liuwei Dihuang Decoction [9, 10], ZiBuPiYin recipe [11–13], Sagacious Confucius’ Pillow Elixir (SCPE) [14, 15] and electroacupuncture therapy [16–19] can significantly improve cognitive function. The above studies found that these treatments can effectively improve the body’s immune function, inhibit inflammation, inhibit apoptosis, improve synaptic plasticity, inhibit excitatory amino acid toxicity, and promote neuron regeneration; thus, it can improve cognitive function. Our team has been devoted to preventing and treating neurodegenerative diseases using traditional Chinese medicine (TCM) for many years. In clinical and experimental studies, SCPE was identified as a TCM with high potential in the clinical treatment of cognitive disorders.

Sagacious Confucius’ Pillow Elixir (SCPE), a classic prescription in TCM, was first described in the classic treatise Prescriptions Worth a Thousand Pieces of Gold (Qianjin Yaofang). Previous studies have indicated that SCPE exerts a considerable effect on CI-related diseases, has a high utilization rate in the clinic, and improves cognitive function in senescence-accelerated prone 8 (SAMP8) mice by inhibiting hippocampal pyroptosis and Aβ accumulation. SCPE comprises Shichangpu (*Acorus tatarinowii*), Yuanzhi (*Polygala tenuifolia* Willd), Guijia (*Chinemys reevesii* (Gray)) and Longgu (Fossilia Ossis Mastodi). Based on the complex characteristics of TCM components, the continuous use of traditional pharmacological methods to evaluate the complex and exact mechanism of action and effectiveness of SCPE for CI therapy is challenging.

For decades, the characteristics of the absorption, distribution, metabolism, excretion and toxicity (ADME/T) of compounds have become critical issues in evaluating the effects or risks of natural compounds on the human body. Because *in vivo* and *in vitro* assessments are expensive and laborious, with the development of computer technology, *in silico* techniques have been widely used to assess these properties [20–23]. Computer virtual screening (VS) technology can help effectively identify active molecules with novel structures, playing a significant role in the early stage of drug development. However, some problems and limitations exist in the application field of model validation technology, model validation techniques, global models and local models [24–26]. In the present study, the toxicity of candidate molecules was analyzed in depth using the Python programming language. Combined with systems pharmacology, the interactions of active components, targets, and diseases, among others, were analyzed and integrated into a multilevel interactive network system. These processes were achieved through the construction and visualization analysis of biological networks at multiple levels. In the present study, the active components of SCPE used to treat CI were screened using systems pharmacology, and the potential molecular mechanism was evaluated using molecular biology tools to support future clinical research on SCPE treatment for CI.

## RESULTS

### Predictive analysis of active components of SCPE and targets involved in treating CI based on systems pharmacology

A CI gene database was obtained by performing an interactive Venn analysis of 6 disease databases: 5205 genes from the GeneCards database, 5033 genes from the OMIM database, 11,316 genes from the CTD database, 42,478 genes from the TTD database, 27,155 genes from the DisGeNet database and 39,768 genes from the TCMSP database, in which 44,564 genes were highly correlated with CI (Supplementary Figure 1). Thirty effective components of SCPE were screened from the related databases—18 species of *Acorus tatarinowii*, 9 species of *Polygala tenuifolia* Willd, 1 species of *Chinemys reevesii* (Gray) and 2 species of Fossilia Ossis Mastodi (Table 1). Combined with the gene set of CI, we conducted predictive integration analysis on the potential targets of 30 active ingredients and identified 376 potential genes involved in the SCPE treatment of CI. Cytoscape software was used for visualization (Supplementary Figure 2). The size of each icon reflects its importance in the overall network. The yellow node represents the disease, the green nodes represent the herbs, the red nodes represent the active compounds, and the blue nodes represent the therapeutic targets obtained after the intersection of SCPE active compound targets and CI-related disease targets.

**Table 1:** Active components of SCPE.

**Herbs (M)**	**Number**	**Components (C)**
*Acorus tatarinowii* (M1)	18	Alloaromadendrene (C1), Caryophyllene oxide (C2), β-Gurjunene (C3), β-Cubebene (C4), 2’-O-Methylisoliquiritigenin (C5), Ledene (C6), α-Longipinene (C7), Aminacrin (C8), Aristolone (C9), Calamendiol (C10), Isocalamendiol (C11), Murolan-3,9(11)-diene-10-peroxy (C12), Patchoulene (C13), spathulenol (C14), α-Panasinsene (C15), (1R,3aS,4R,6aS)-1,4-bis (3,4-dimethoxyphenyl)-1,3,3a,4,6,6a-hexahydrofuro (4,3-c) furan (C16), Cycloartenol (C17), α-cedrene (C18)
*Polygala tenuifolia* Willd (M2)	9	5,6,7-Trimethoxycoumarin (C19), Yajeine (C20), Norhyoscyamine (C21), Onjixanthone I (C22), Euxanthone (C23), 1,6-Dihydroxy-3,7-dimethoxyxanthone (C24), Gentisin (C25), Perlolyrine (C26), Frutinone A (C27)
*Chinemys reevesii* (Gray) (M3)	1	Cholesterol (C28)
Fossilia Ossis Mastodi (M4)	2	Calcium carbonate (C29), Calcium phosphate (C30)

### GO and KEGG pathway enrichment analyses

**Figure 1:**
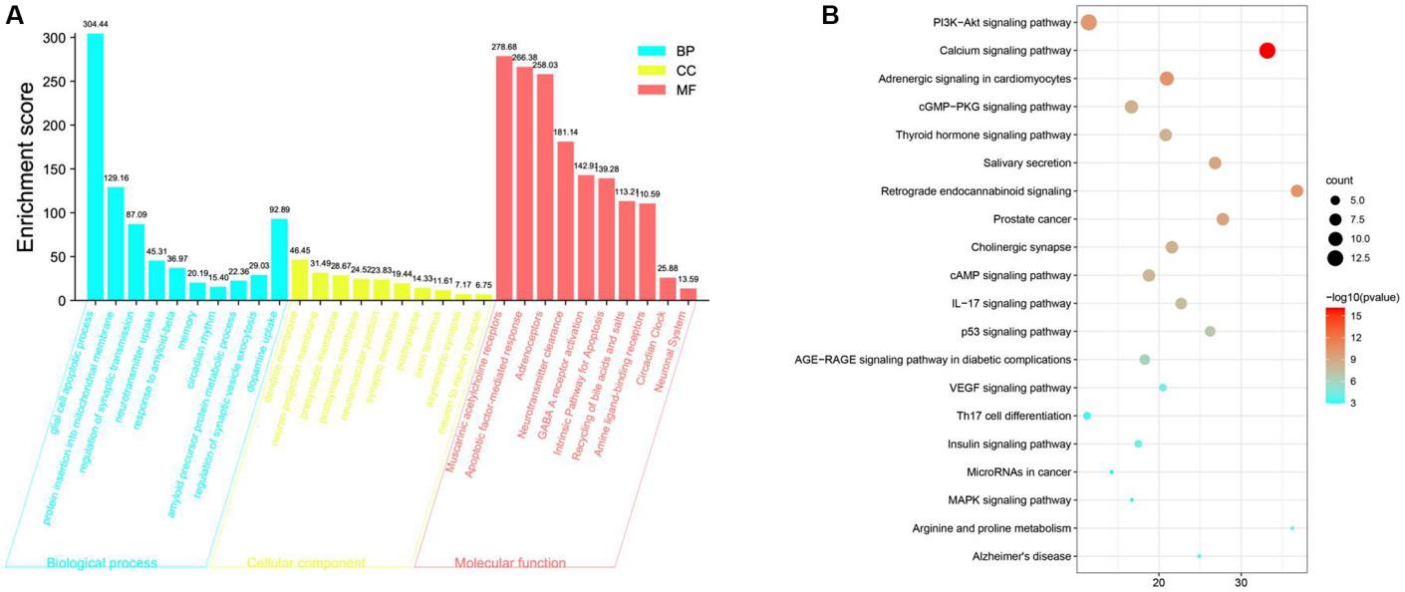
**Gene Ontology (GO) and KEGG pathway enrichment analyses of targets of SCPE to treat cognitive impairment (CI).** (**A**) Gene Ontology (GO) enrichment results. (**B**) KEGG pathway enrichment results.

The underlying biological processes (BPs), cellular components (CCs) and molecular functions (MFs) of the 376 target genes were identified by performing Gene Ontology (GO) enrichment analysis. By setting the filter to an adjusted *p* value < 0.05 and *q*-value < 0.05, 1711 significantly enriched GO terms were identified. The first 30 terms are shown in Figure 1A. GO terminology suggests that these target genes play essential roles in mitochondrial regulation of intersynaptic neurotransmitter transport and intracellular metabolic waste removal, affecting cognitive function (Figure 1A).

KEGG enrichment analysis was performed to identify the pathways in which the target genes were enriched. The data were filtered using the criteria of an adjusted *P* value < 0.05 and *q*-value < 0.05. A bubble chart of the 20 most significant KEGG pathways is shown in Figure 1B; the PI3K/AKT pathway is one of the most important pathways (Figure 1B).

### Pharmacological and toxicological analysis and molecular docking of active components with proteins in the PI3K/AKT/mTOR signaling pathway

#### Analysis of the basic parameters of the active components

The results above showed that among the 20 signaling pathways with gene enrichment, the PI3K/AKT/mTOR signaling pathway was the most critical. Twenty-three active components of SCPE were found to interact with the PI3K/AKT/mTOR signaling pathway. The basic information of these compounds is shown in Table 2. The basic parameters of these 23 monomers were further evaluated using absorption, distribution, metabolism, excretion and toxicity (ADME/T) prediction software. Most of the 23 monomers were safe and suitable for drug development. The specific results are shown in Table 2 and Supplementary Figure 3.

**Table 2:** Basic information of the 23 active components of SCPE.

**SCPE Herb**	**No.**	**Monomer**	**OB**	**DL**	**BBB**	**MW**	**PL**	**PT**
Acorus tatarinowii	C1	Alloaromadendrene	54.04	0.10	2.07	204.39	5000	V
	C2	Caryophyllene oxide	32.67	0.13	1.76	220.39	5000	V
	C3	β-Gurjunene	51.36	0.10	2.07	204.39	1190	IV
	C4	β-Cubebene	32.81	0.11	2.02	204.39	5000	V
	C5	2’-O-Methylisoliquiritigenin	75.86	0.17	−0.16	270.3	3000	V
	C6	()-Ledene	51.84	0.10	2.16	204.39	5000	V
	C7	α-Longipinene	57.47	0.12	2.05	204.39	3700	V
	C9	Aristolone	45.31	0.13	1.54	218.37	1870	IV
	C10	Calamendiol	61.13	0.11	0.67	236.39	3900	V
	C11	Isocalamendiol	57.63	0.11	0.74	238.41	3900	V
	C12	Murolan-3,9(11)-diene-10-peroxy	36.72	0.11	1.04	236.39	4300	V
	C13	Patchoulene	49.06	0.11	2.17	204.39	5000	V
	C14	Spathulenol	81.61	0.12	1.55	220.39	3900	V
	C15	α-Panasinsene	56.77	0.12	2.11	204.39	5000	V
	C18	α-Cedrene	55.56	0.10	2.16	204.39	5000	V
	C19	5,6,7-Trimethoxycoumarin	32.54	0.12	0.54	236.24	3800	V
	C20	Yajeine	56.8	0.13	0.79	212.27	500	IV
	C22	Onjixanthone I	79.16	0.30	0.04	302.3	3800	V
Polygala tenuifolia Willd	C23	Euxanthone	92.98	0.16	0.15	228.21	2991	IV
	C24	1,6-Dihydroxy-3,7-dimethoxyxanthone	89.65	0.27	−0.03	288.27	3800	V
	C25	Gentisin	64.06	0.21	−0.09	258.24	4000	V
	C26	Perlolyrine	65.95	0.27	0.15	264.3	600	IV
	C27	Frutinone A	65.90	0.34	0.46	264.24	322	IV

Abbreviations: OB: oral bioavailability; DL: drug-likeness; BBB: permeability across the blood–brain barrier; MW: molecular weight; PL: predicted LD50 (mg/kg); PT: predicted toxicity class. I: fatal if swallowed (LD50 ≤ 5); II: fatal if swallowed (5 < LD50 ≤ 50); III: toxic if swallowed (50 < LD50 ≤ 300), IV: harmful if swallowed (300 < LD50 ≤ 2000); V: may be harmful if swallowed (2000 < LD50 ≤ 5000); VI: nontoxic (LD50 > 5000).

### PPI network construction and molecular docking analysis of transcription factors

The PPI network was constructed using the STRING database, and Cytoscape was used to visualize the PPI network (Supplementary Figure 4). The PPI network was used to analyze all potential target genes of SCPE. The results revealed 84 nodes and 314 edges (Supplementary Figure 3), representing the interactions between proteins and illustrating their functions.

The 5 most significant genes, TP53, CND1, EP300, CASP3 and RAC1, were selected for molecular docking. Molecular docking of 23 active ingredients in SCPE with core targets was conducted, and the top 5 docking results of the binding energy of 5 core targets were obtained (Table 3). The greater was the absolute value of affinity, the stronger was the binding ability between the receptor and ligand.

**Table 3:** Top 5 docking results of SCPE intervention in the binding energy of each core target of CI.

**Target (PDB ID)**	**Active Compound**	**Binding energy/kcal/mol**	**Herb**
CCND1 (6P8F) [27]	2’-O-Methylisoliquiritigenin	−6.0	*Acorus tatarinowii*
TP53 (6GGA) [28]	2’-O-Methylisoliquiritigenin	−6.2	*Acorus tatarinowii*
CASP3 (6CKZ) [29]	Perlolyrine	−6.4	*Polygala tenuifolia* Willd
RAC1 (5O33) [30]	Onjixanthone I	−7.0	*Polygala tenuifolia* Willd
EP300 (6PGU) [31]	Gentisin	−7.0	*Polygala tenuifolia* Willd

### Docking of CCND1 with 2’-O-methylisoliquiritigenin

**Figure 2:**
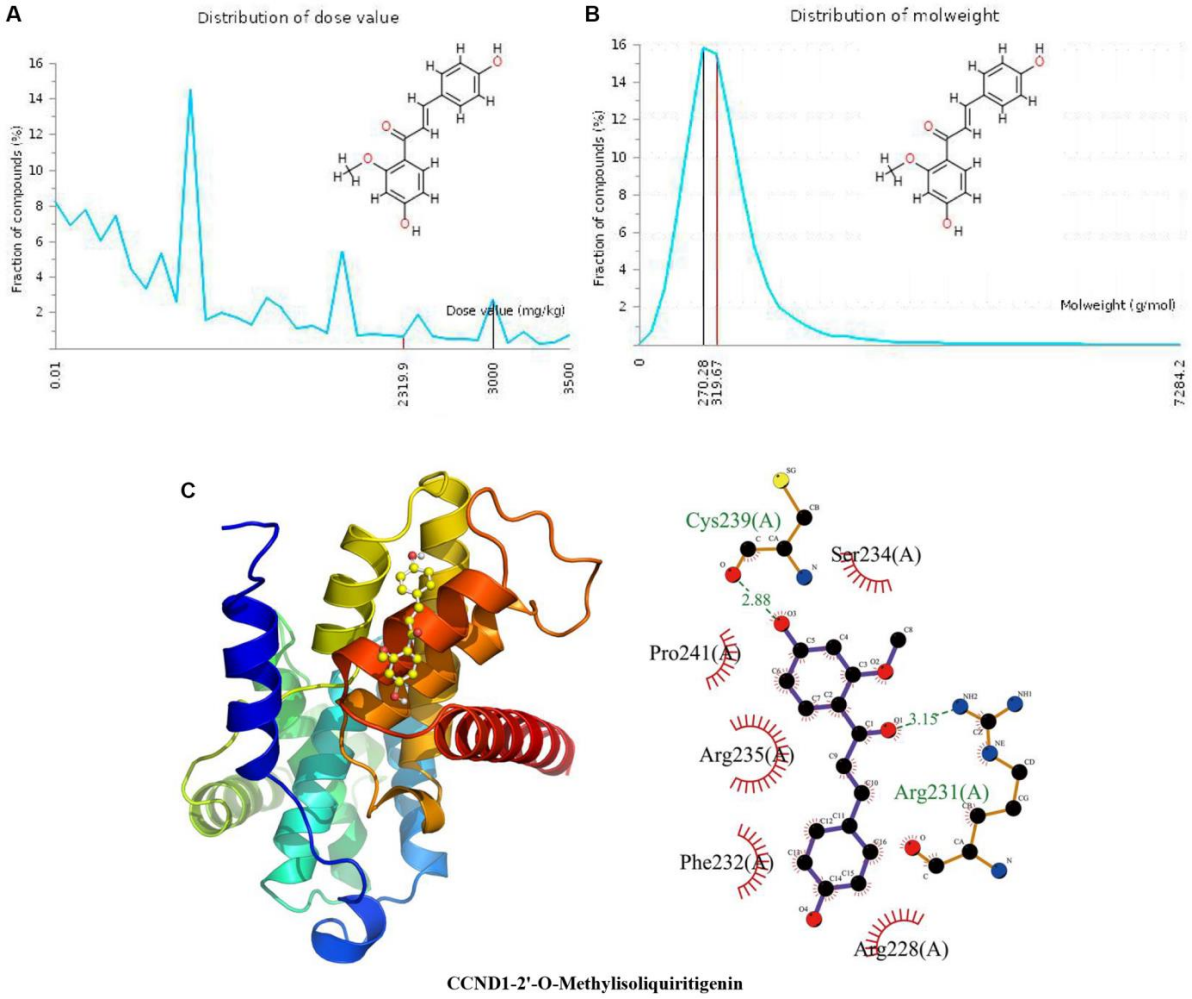
**Distribution of the 2’-O-methylisoliquiritigenin dose value and molecular weight and docking with CCND1.** (**A**) Distribution of the dose value of 2’-O-methylisoliquiritigenin. (**B**) Distribution of the molecular weight of 2’-O-methylisoliquiritigenin. (**C**) Molecular docking of 2’-O-methylisoliquiritigenin with the CCND1 protein.

The distribution of the calculated LD50 values is shown in Figure 2A. The mean of the dataset is shown in red (2319.9 mg/kg), and the predicted median lethal dose of the input 2’-O-methylisoliquiritigenin is shown in black (3000 mg/kg). Figure 2B indicates the MW distribution of 2’-O-methylisoliquiritigenin in our dataset. The mean MW is indicated as a red line (319.67 g/mol), whereas the MW of the input compound is indicated as a black line (270.28 g/mol). These results indicate that 2’-O-methylisoliquiritigenin is a candidate compound with good properties. In Figure 2C, the calculated binding pose of ligands of 2’-O-methylisoliquiritigenin to the CCND1 protein was −6.0 kcal/mol, demonstrating a good binding effect. 2’-O-Methylisoliquiritigenin interacts with the CCND1 protein primarily by forming hydrogen bonds with Arg231 (A) and Cys2239 (A) with lengths of 3.15 Å and 2.88 Å, respectively, through hydrophobic interactions. Hydrophobic bonds often play a key role in the formation of space-folded biofilms of protein polypeptide chains, the interaction between macromolecules, and the catalysis of enzymes on substrate molecules. It is hydrophobic with Arg228 (A), Phe232 (A), Arg235 (A), Pro241 (A), and Ser234 (A).

### Docking of TP53 with 2’-O-methylisoliquiritigenin

**Figure 3:**
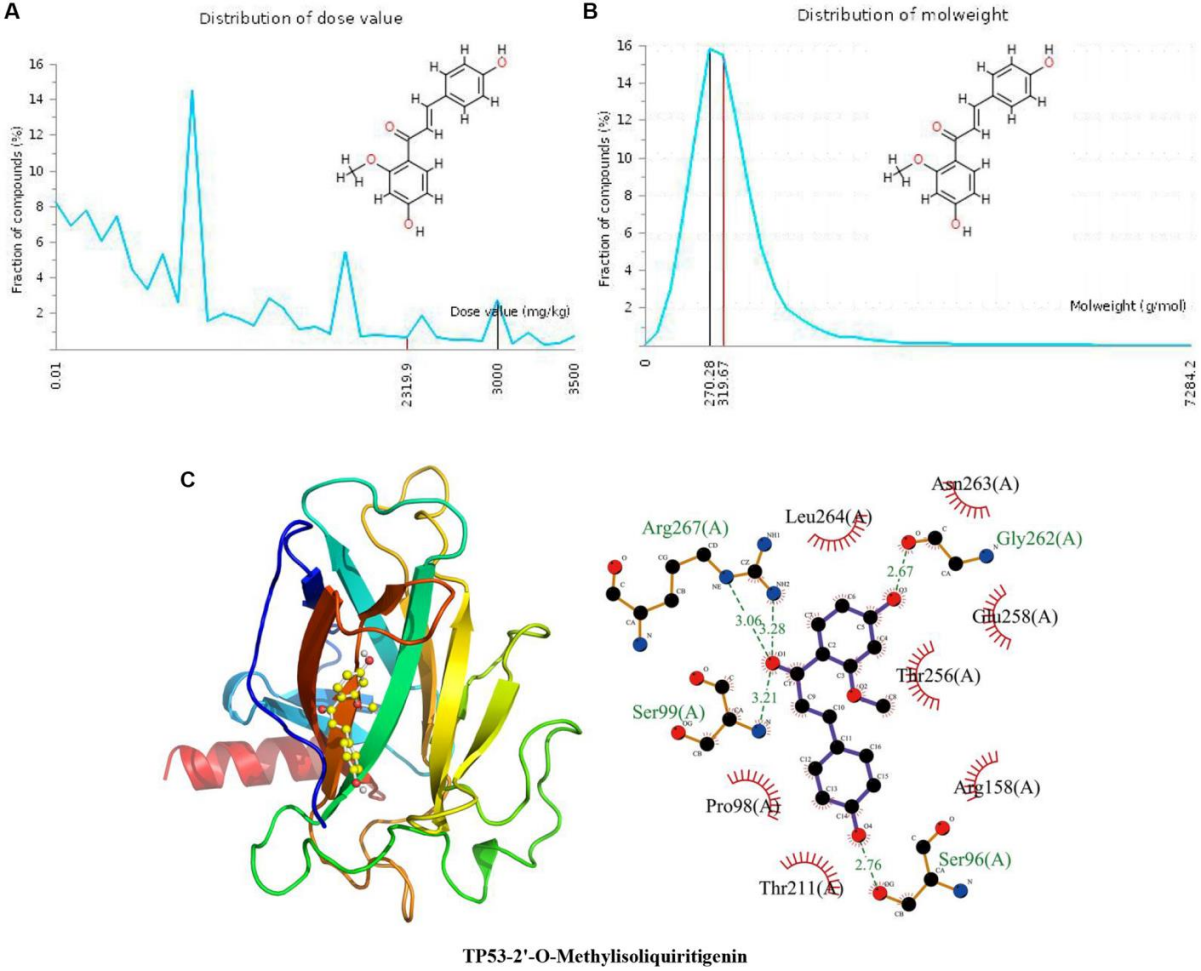
**Distribution of the 2’-O-methylisoliquiritigenin dose value and molecular weight and docking with TP53.** (**A**) Distribution of the dose value of 2’-O-methylisoliquiritigenin. (**B**) Distribution of the molecular weight of 2’-O-methylisoliquiritigenin. (**C**) Molecular docking of 2’-O-methylisoliquiritigenin with the TP53 protein.

The specific properties of 2’-O-methylisoliquiritigenin are shown in the above results of docking with CCND1 and in Figure 3A and 3B. In Figure 3C, the calculated binding pose of ligands of 2’-o-methylisoliquiritigenin to the TP53 protein was −6.2 kcal/mol, demonstrating a good binding effect. 2’-O-Methylisoliquiritigenin interacts with the TP53 protein mainly through the formation of hydrogen bonds with Ser96 (A), Gly262 (A), Arg267 (A) and Ser99 (A) and hydrophobic interactions. The length of the hydrogen bond was 2.76 Å with Ser96 (A), 2.67 Å with Gly262 (A), 3.28 Å and 3.06 Å with Arg267 (A), and 3.21 Å with Ser99 (A). 2’-O-Methylisoliquiritigenin is hydrophobic with Thr211 (A), Pro98 (A), Leu264 (A), Asn263 (A), Glu258 (A), Thr256 (A) and Arg158 (A).

### Docking of CASP3 with perlolyrine

**Figure 4:**
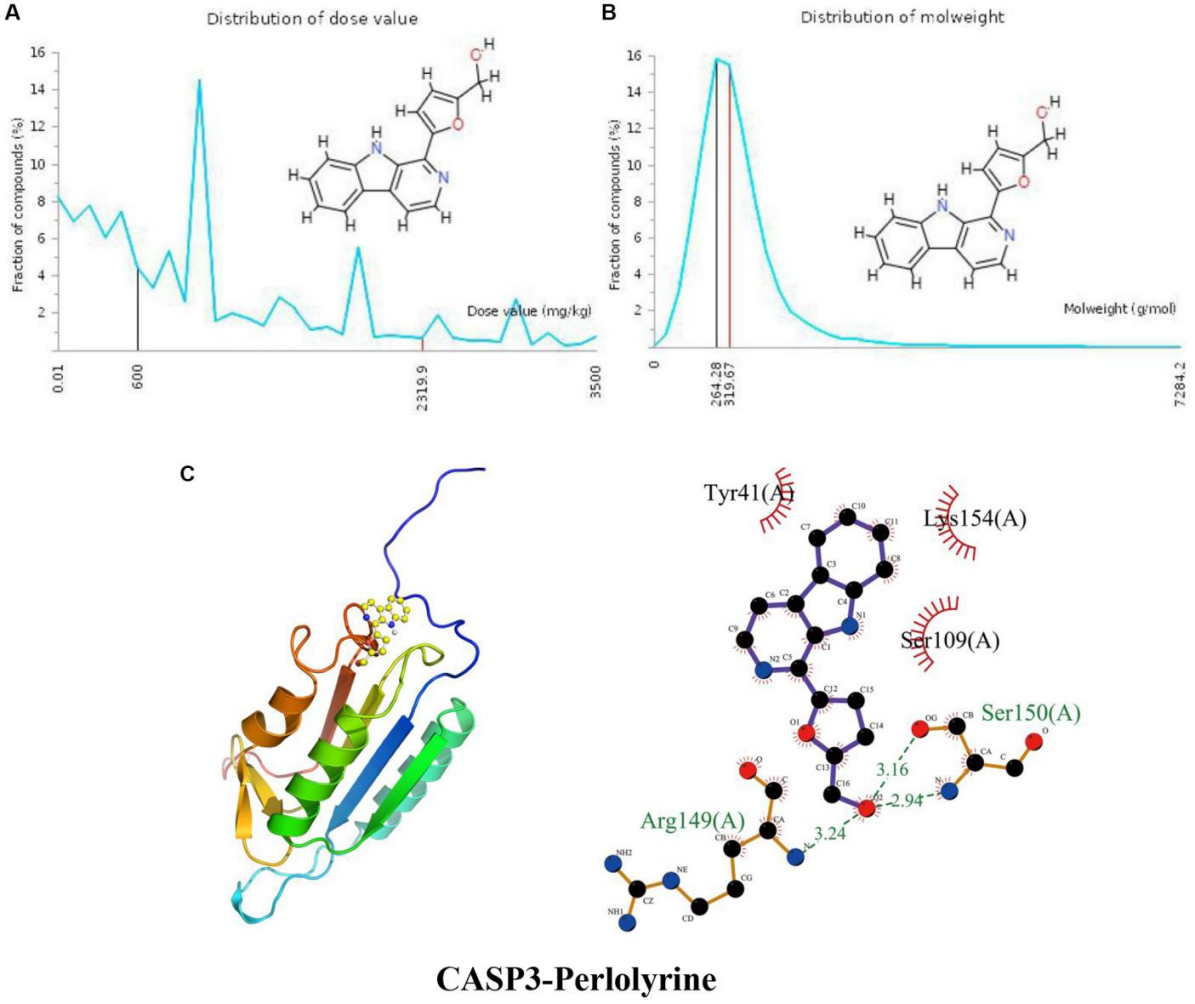
**Distribution of the perlolyrine dose value and molecular weight and docking with CASP3.** (**A**) Distribution of the dose value of Perlolyrine. (**B**) Distribution of the molecular weight of perlolyrine. (**C**) Molecular docking of perlolyrine with the CASP3 protein.

The distribution of the calculated LD50 values is shown in Figure 4A. The mean of the dataset is shown in red (2319.9 mg/kg), and the predicted median lethal dose of the input perlolyrine is shown in black (600 mg/kg). Figure 4B indicates the MW distribution of perlolyrine in our dataset. The mean MW is indicated as a red line (319.67 g/mol), whereas the MW of the input compound is indicated as a black line (264.28 g/mol). These results indicate that perlolyrine is a toxic candidate compound to a certain extent. In Figure 4C, the calculated binding pose of the ligands of perlolyrine to the CASP3 protein was −6.4 kcal/mol, demonstrating a good binding effect. Perlolyrine interacts with CASP3 mainly through hydrogen bonds with Arg149 (A) and Ser150 (A) with lengths of 3.24 Å, 3.16 Å and 2.94 Å, respectively, and hydrophobic interactions. Perlolyrine is hydrophobic with Ser109 (A), Lys154 (A), and Tyr41 (A).

### Docking of RAC1 with Onjixanthone I

**Figure 5:**
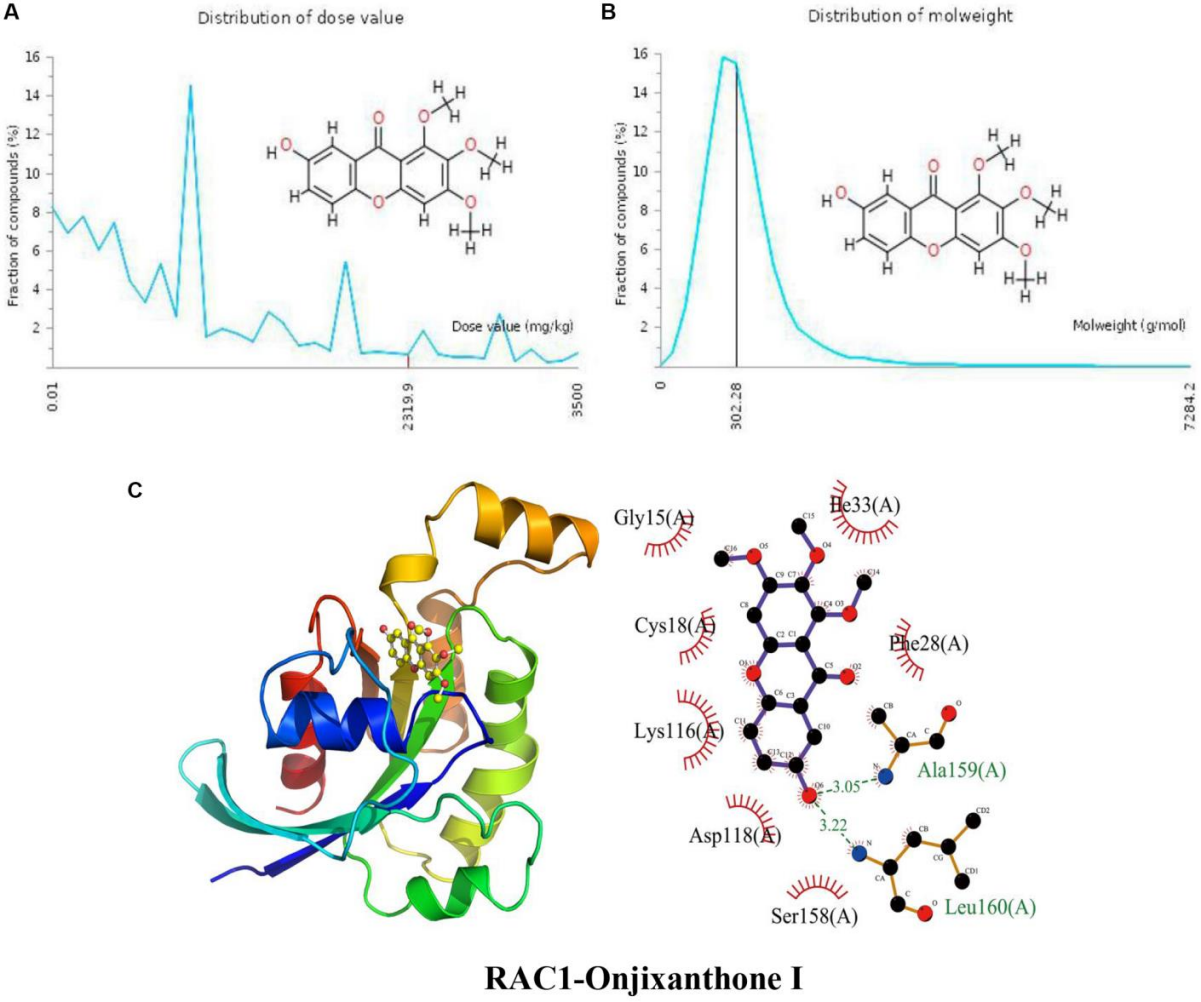
**Distribution of the Onjixanthone I dose value and molecular weight and docking with RAC1.** (**A**) Distribution of the dose value of Onjixanthone I. (**B**) Distribution of the molecular weight of Onjixanthone I. (**C**) Molecular docking of Onjixanthone I with the RAC1 protein.

The distribution of the calculated LD50 values is shown in Figure 5A. The mean of the dataset is shown in red (2319.9 mg/kg). Figure 5B indicates that the MW of the input Onjixanthone I is indicated as a black line (302.28 g/mol). These results indicate that Onjixanthone I is a candidate toxic compound to a certain extent. The calculated binding pose of the ligands of Onjixanthone I to RAC1 protein was −7.0 kcal/mol, indicating that Onjixanthone I had a good binding effect (Figure 5C). Onjixanthone I interacts with the RAC1 protein mainly through the formation of hydrogen bonds and hydrophobic interactions and forms hydrogen bonds with Ala159 (A) and Leu160 (A) with lengths of 3.05 Å and 3.22 Å, respectively, Ser158 (A), Asp118 (A), Lys116 (A), Cys18 (A), Gly15 (A), Ile33 (A), and Phe28 (A).

### Docking of EP300 with gentisin

**Figure 6:**
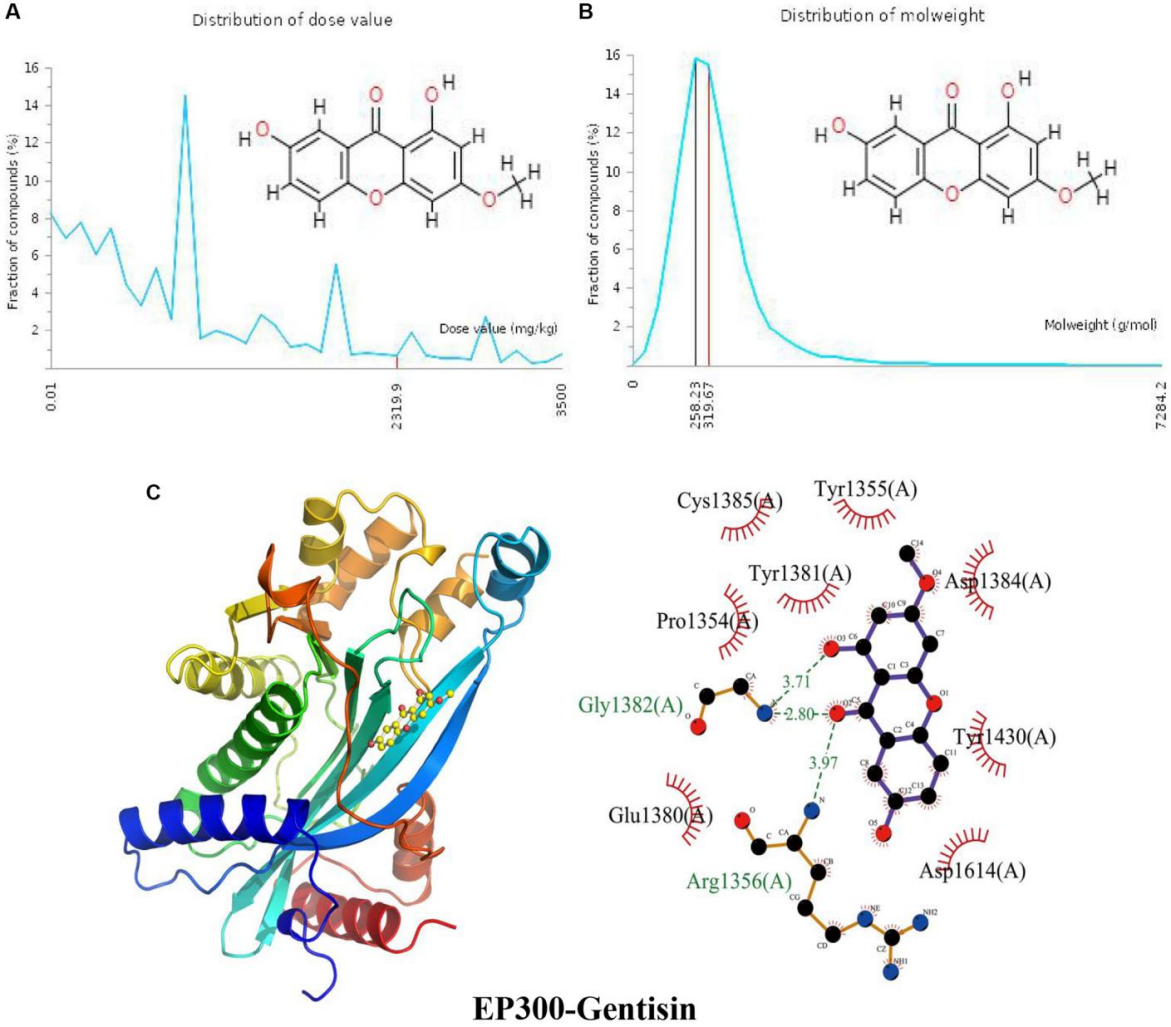
**Distribution of the gentisin dose value and molecular weight and docking with EP300.** (**A**) Distribution of the dose value of gentisin. (**B**) Distribution of the molecular weight of gentisin. (**C**) Molecular docking of gentisin with the EP300 protein.

The distribution of the calculated LD50 values is shown in (Figure 6A). The mean of the dataset is shown in red (2319.9 mg/kg). Figure 6B indicates the MW distribution of gentisin in our dataset. The mean MW is indicated as a red line (319.67 g/mol), whereas the MW of the input gentisin is indicated as a black line (258.23 g/mol). These results indicate that gentisin is a candidate compound with good properties. The calculated binding pose of ligands of gentisin to the EP300 protein was −7.0 kcal/mol, demonstrating a good binding effect (Figure 6C). Gentisin interacts with the EP300 protein primarily by forming hydrogen bonds with Arg1356 (A) and Gly1382 (A) with lengths of 3.97 Å, 2.80 Å and 3.71 Å, respectively, and hydrophobic interactions. Gentisin is also hydrophobic with Glu1380 (A), Pro1354 (A), Tyr1381 (A), Cys1385 (A), Tyr1355 (A), Asp1384 (A), Tyr1430 (A) and Asp1614 (A).

The molecular docking results revealed that the active ingredients with better docking results with the core target were 2’-O-methylisoliquiritigenin, perlolyrine, gentisin and onjixanthone I. All the compounds used to treat CI were derived from phytomedicine: *Acorus tatarinowii* and *Polygala tenuifolia* Willd.

### Effect of SCPE on the mouse CI model

#### MWM evaluation of the effects of SCPE on CI

##### Acquisition test results

In the first part of the acquisition trial, the target latency and total swimming distance did not differ between the groups, suggesting that CI model (SAMP8) mice had normal visual and motor functions similar to those of the control group (SAMR1); thus, the animals were used in subsequent studies. The learning curve generated from the 4-day acquisition test showed spatial learning and memory impairments in CI model mice. In other words, the curve of control mice was steeper, indicating faster task acquisition, while the curve of CI model mice was shallower, indicating difficulty in task acquisition. The learning curves of the groups treated with the 3 doses of SCPE were the same as those of the normal control group, indicating that the drug improved the impaired cognitive function of CI model mice. The results of the statistical analysis are summarized below.

Compared with the normal control group, the target latency of CI model mice (F = 21.39; *P* < 0.0001) and total swimming distance (F = 14.5, *P* < 0.0001) increased significantly. Two-way ANOVA showed significant differences in the target latency (F = 10.55; *P* < 0.0001) and total swimming distance (F = 17.15; *P* < 0.0001) between the SCPE treatment group and model group. The goal latency of the 9.2 g/kg SCPE-treated group on day 3 (*P* < 0.05) and total swimming distance on day 2 (*P* < 0.05) were significantly higher than those of the 2.3 g/kg SCPE-treated group. No significant differences in the target goal latency or total swimming distance were observed between the other groups.

### Probe test results

In the probe test, the residence time of the CI mice in the target quadrant (*P* < 0.01) and number of platform crossings (*P* < 0.01) were significantly decreased compared with those of the control group, consistent with the trend observed for the first part of the acquisition test results.

**Figure 7:**
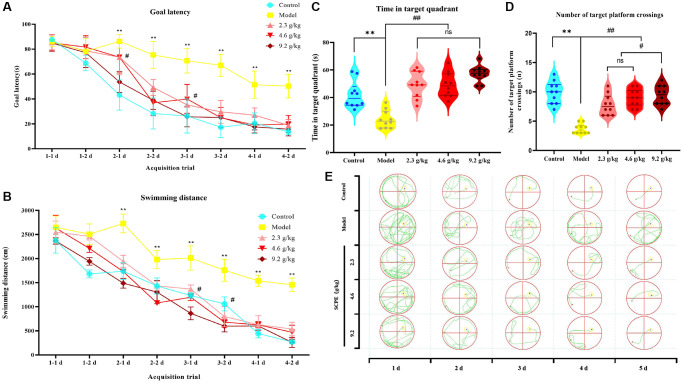
**Effects of SCPE on CI in model mice measured using the Morris water maze test.** (**A**, **B**) Acquisition tests. (**A**) Goal/escape latency was measured twice per day. (**B**) Total swimming distance measured twice per day. SCPE was administered orally at doses of 2.3 g/kg, 4.6 g/kg and 9.2 g/kg. The MWM test was performed 2 h after oral administration. The data are presented as means ± SD (*n* = 10). ^*^P < 0.05 and ^**^P < 0.01 compared with the control group. ^#^*P* < 0.05 compared with the model group. (**C**, **D**) Probe test. (**C**) Time spent in the target/first quadrant; (**D**) Number of platform crossings. The data are presented as the means ± SD (*n* = 10). ^**^P < 0.01 compared with the control group. ^#^*P* < 0.05 compared with the model group. (**E**) Real swimming tracks of mice in each group in the MWM.

Compared with the model group, the SCPE-treated groups (2.3, 4.6 and 9.2 g/kg) showed significantly increased residence times in the target quadrant (*P* < 0.01) and number of platform crossings (*P* < 0.01). The SCPE-treated groups and control group showed no significant difference in the residence time in the target quadrant (*P* > 0.05). Compared with the groups treated with 2.3 g/kg and 4.6 g/kg of SCPE, the number of platform crossings by the 9.2 g/kg SCPE-treated group was increased (*P* < 0.05) (Figure 7).

### Neuropathological results

#### HE staining

**Figure 8:**
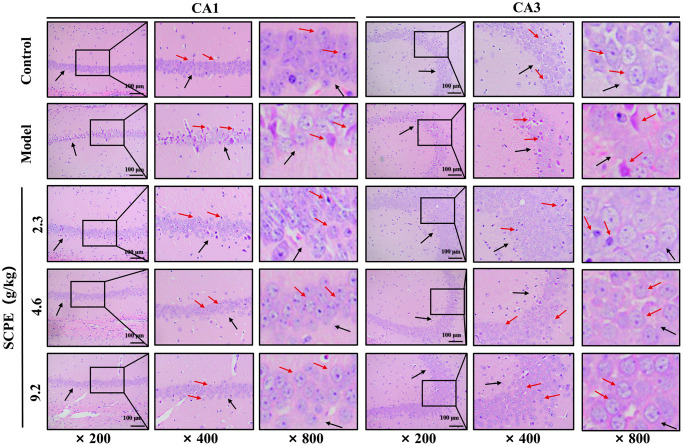
**HE staining of the hippocampal CA1 and CA3 regions (*n* = 3).** The black arrows show the arrangement of pyramidal cells and intercellular space. The red arrows show the morphology of the neuron.

Conventional slices of mouse hippocampal tissues were stained with HE and viewed at high (×800, ×400) and low (×200) magnification (Figure 8). In the control group, the morphology and structure of neurons in the hippocampal CA1-CA3 area were normal, the cell bands were tightly arranged, and the number of neuron layers was greater. The number of pyramidal cells was greater than that in the other groups. The nuclei were large and round, the nucleoli were clear, the staining was shallow, the cytoplasm was rich, the staining was uniform, no obvious abnormal pathological changes, such as cell lysis or nuclear pyknosis, were observed, the nuclear membrane was clear, and a few normal glial cells were observed. In the model group, the numbers of normal neurons in both the CA1 and CA3 areas were obviously decreased, and abnormal pathological manifestations, such as cell necrosis and degeneration, were significant. The pyramidal cells were irregular in shape, decreased in volume, and scattered in arrangement, and showed obvious nuclear pyknosis and glial proliferation. These pathological changes were improved in the 2.3 g/kg SCPE-treated group compared with the model group, but to a lesser extent. The pathological changes in both the hippocampal CA1 and CA3 regions were significantly improved in the 4.6 g/kg and 9.2 g/kg SCPE-treated groups compared with those in the model group. The number of neurons was significantly increased, the cells were arranged relatively neatly and tightly, and the number of degenerating cells was significantly reduced. The number of pyramidal cells increased significantly, the degree of nuclear pyknosis was reduced, the nuclear membrane was clear, and a few normal glial cells were observed.

### Toluidine blue staining

**Figure 9:**
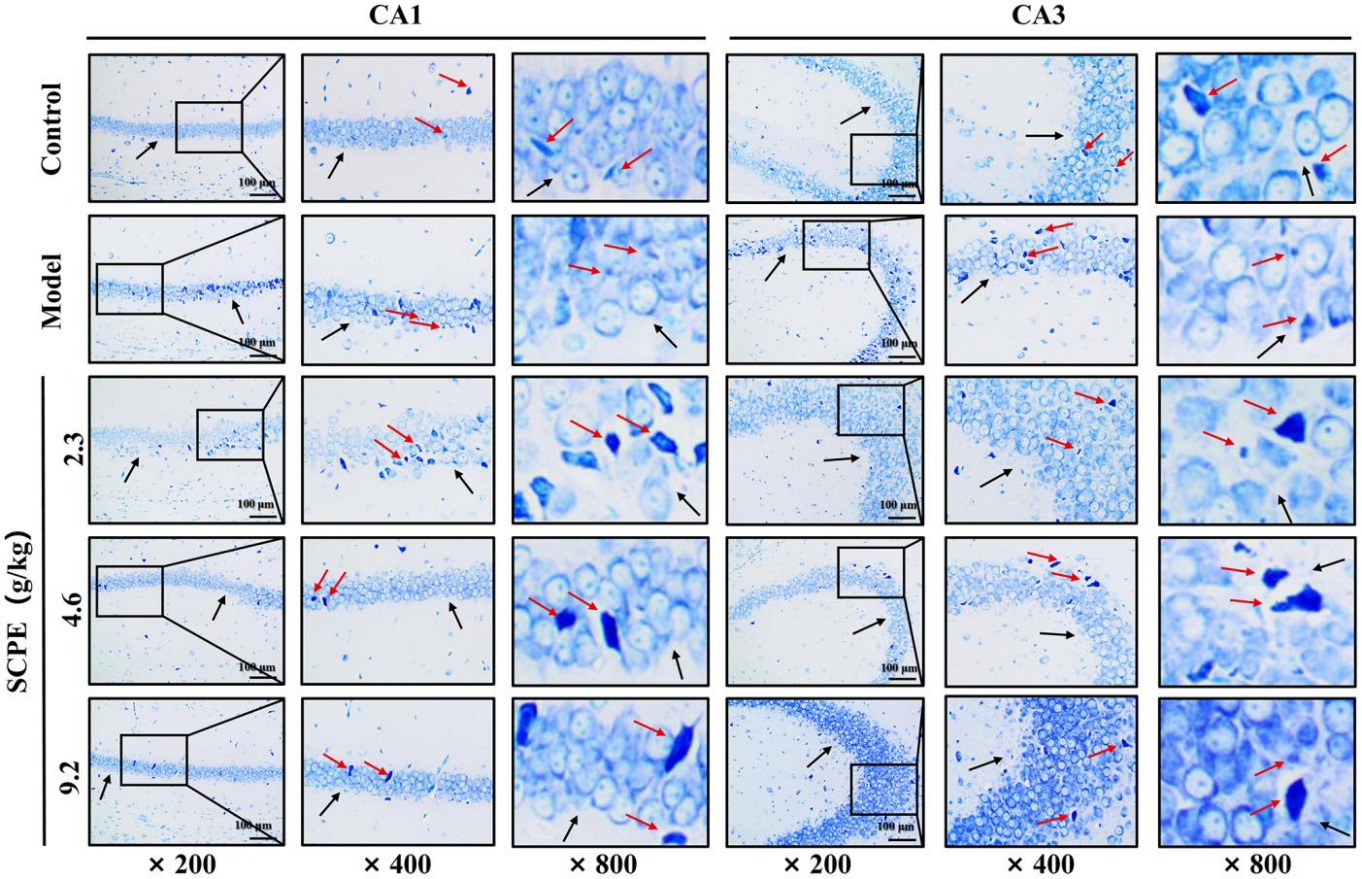
**Toluidine blue staining in the hippocampal CA1 and CA3 regions of each group (*n* = 3).** The black arrows indicate the arrangement of pyramidal cells and the basic morphology of neurons in the CA1 and CA3 regions of the hippocampus. The red arrows represent the Nissl body.

Nissl bodies in the hippocampal CA1 and CA3 regions were observed under a microscope (Figure 9). The number and content of Nissl bodies were abundant in the control group. In the model group, Nissl bodies were granular or disintegrated, disappeared and were small and scattered. Compared with the model group, the number and volume of Nissl bodies increased in the 3 SCPE-treated groups. These results suggested that SCPE improved the metabolic level of neurons in the hippocampus of CI mice.

### Transmission electron microscopy (TEM)

**Figure 10:**
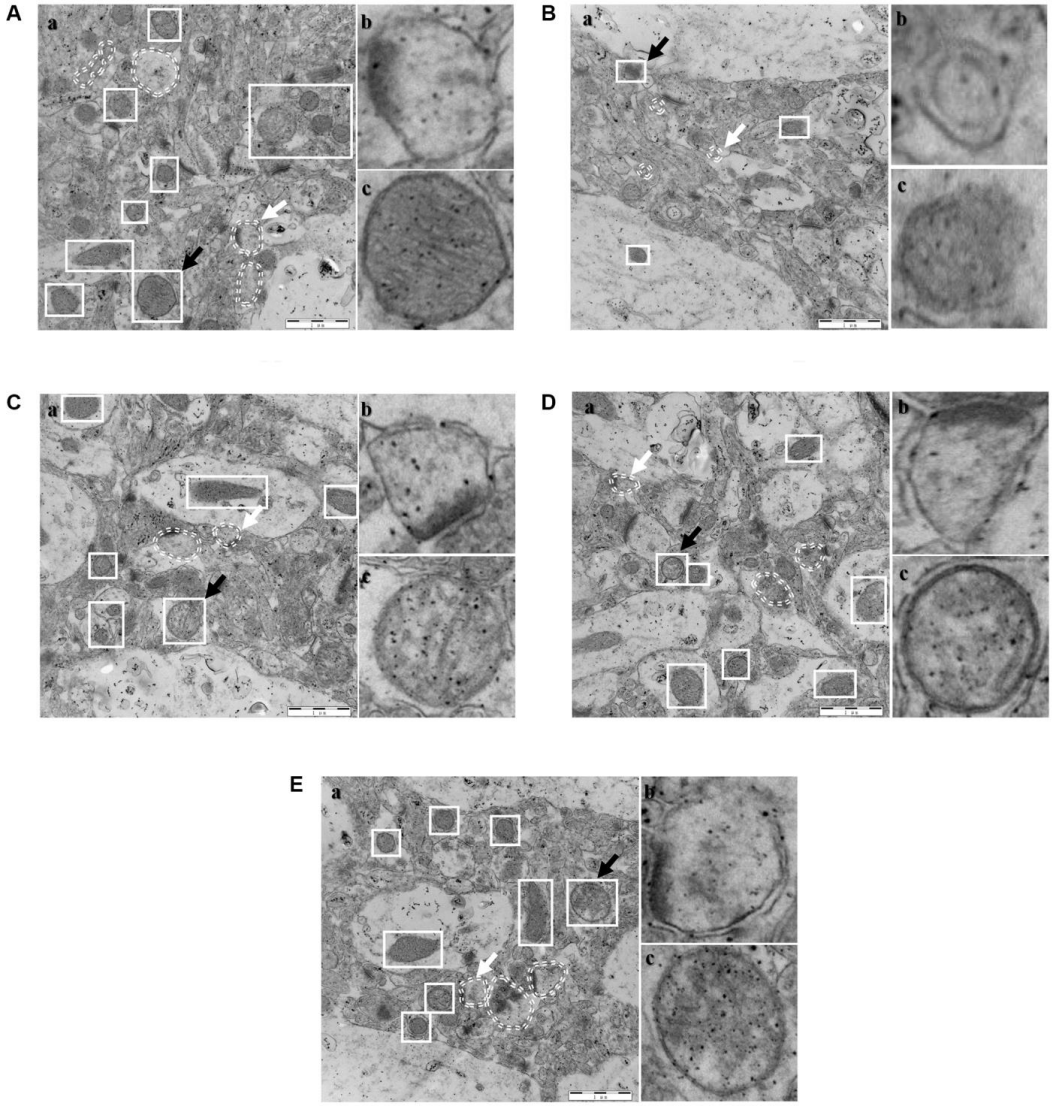
**Electron microscope sections of the hippocampi of mice (*n* = 3).** (**A**) Control; (**B**) Model; (**C**) SCPE 2.3 g/kg; (**D**) SCPE 4.6 g/kg; (**E**) SCPE 9.2 g/kg. (**a**) TEM detection; (**b**) enlarged autophagosomes; (**c**) enlarged mitochondria. The white elliptical dotted lines and white arrows indicate enlarged autophagosomes. The white solid square lines and black arrows indicate enlarged mitochondria. Bars: 1 μm.

Mitochondria and autophagosomes in the hippocampus were the focus of TEM detection. Typical autophagosomes in each group were represented by white elliptical dotted lines, and mitochondria were enclosed by white solid square lines (Figure 10A; the results from each group are shown). The lysosomes (white arrow) and mitochondria (black arrow) in each group were magnified (Figure 10B and 10C) and indicated on the right side for each group. A specific number of autophagosomes was observed in the control group and presented a typical structure of the bilayer membrane and contents and no obvious outflow of contents. The mitochondrial membrane structure was intact, and the cristae were intact. In the model group, the number of autophagosomes was reduced, and the volume of autophagosomes with a bilayer membrane structure was smaller. The mitochondrial membrane structure was incomplete, the cristae were disrupted, and the volume was substantially reduced. The SCPE-treated groups showed a significant reversal of the changes in autophagosomes and mitochondria in the CI model, primarily manifested as increases in the number and volume of lysosomes; the structure and contents of bilayer membranes were also markedly changed, and some of the pathological changes in the contents were mitigated (Figure 10D, 10E). The number of mitochondria was significantly increased, the membrane structure was intact, and the cristae were intact, suggesting that SCPE promoted mitochondrial metabolism in CI mice and hippocampal autophagy.

### Cerebrospinal fluid microdialysis results

#### Linear relationship between 5-HIAA and 5-HT

The standard products of the two neurotransmitters at different concentrations were injected directly for detection. The peak area was taken as the Y value, and the neurotransmitter concentration was taken as the x value. The linear regression equation of the two transmitters, 5-HIAA and 5-HT, was obtained (Table 4). The linear equation demonstrated a good linear relationship between 7.8125 and 500 pg/mL.

**Table 4:** Linear equations of 5-HT and 5-HIAA (x ± s).

**Neurotransmitter**	**Equation of standard curve**	**R**
5-HIAA	y = 0.26794 × −0.14963	0.999
5-HT	y = 0.14419 × +1.14817	0.998

### Determination of the 5-HIAA and 5-HT concentrations in mice

**Figure 11:**
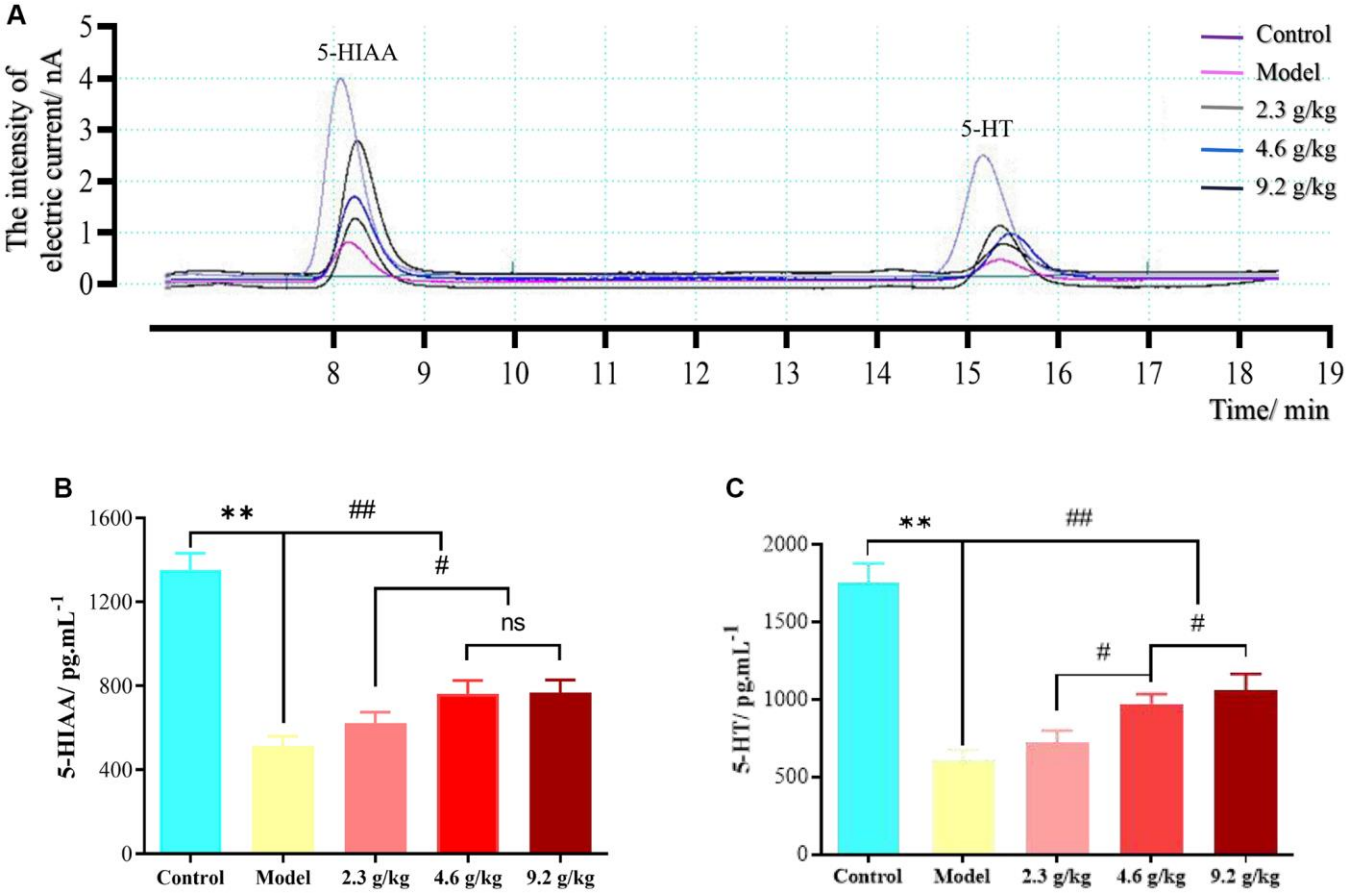
**Cerebrospinal fluid microdialysis results for the hippocampal CA1 region in each group.** (**A**) Chromatogram of neurotransmitters. (**B**) 5-HIAA levels in each group. (**C**) 5-HT levels in each group. The data are presented as means ± SD (*n* = 3). ^**^P < 0.01 and ^##^*P* < 0.01 compared with the model group; ^#^*P* < 0.05 compared with the 2.3 g/kg or 4.6 g/kg group. Abbreviation: ns: not significant.

Microdialysis of mouse cerebrospinal fluid was measured in the hippocampal CA1 region of mice, and a typical chromatogram is shown in Figure 11. The two analytes could be completely separated within 20 min. The concentrations of 5-HT and 5-HIAA in the cerebral dialysate of mice in each group are shown in Table 4.

Compared with the control group, the concentrations of the two neurotransmitters in the dialysate of the model group were significantly decreased (*P* < 0.01). Compared with the model group, the levels of 5-HT and 5-HIAA in the dialysate of mice in the 4.6 g/kg and 9.2 g/kg groups were significantly increased (*P* < 0.01). Compared with the 2.3 g/kg group, the levels of 5-HT and 5-HIAA in the dialysate of both the 4.6 g/kg and 9.2 g/kg groups were significantly increased (*P* < 0.01). Compared with the 4.6 g/kg group, the level of 5-HT in the dialysate of the 9.2 g/kg group was significantly increased (*P* < 0.01).

### Effects of SCPE on the expression of proteins in the PI3K/AKT/mTOR signaling pathway in CI mice

**Figure 12:**
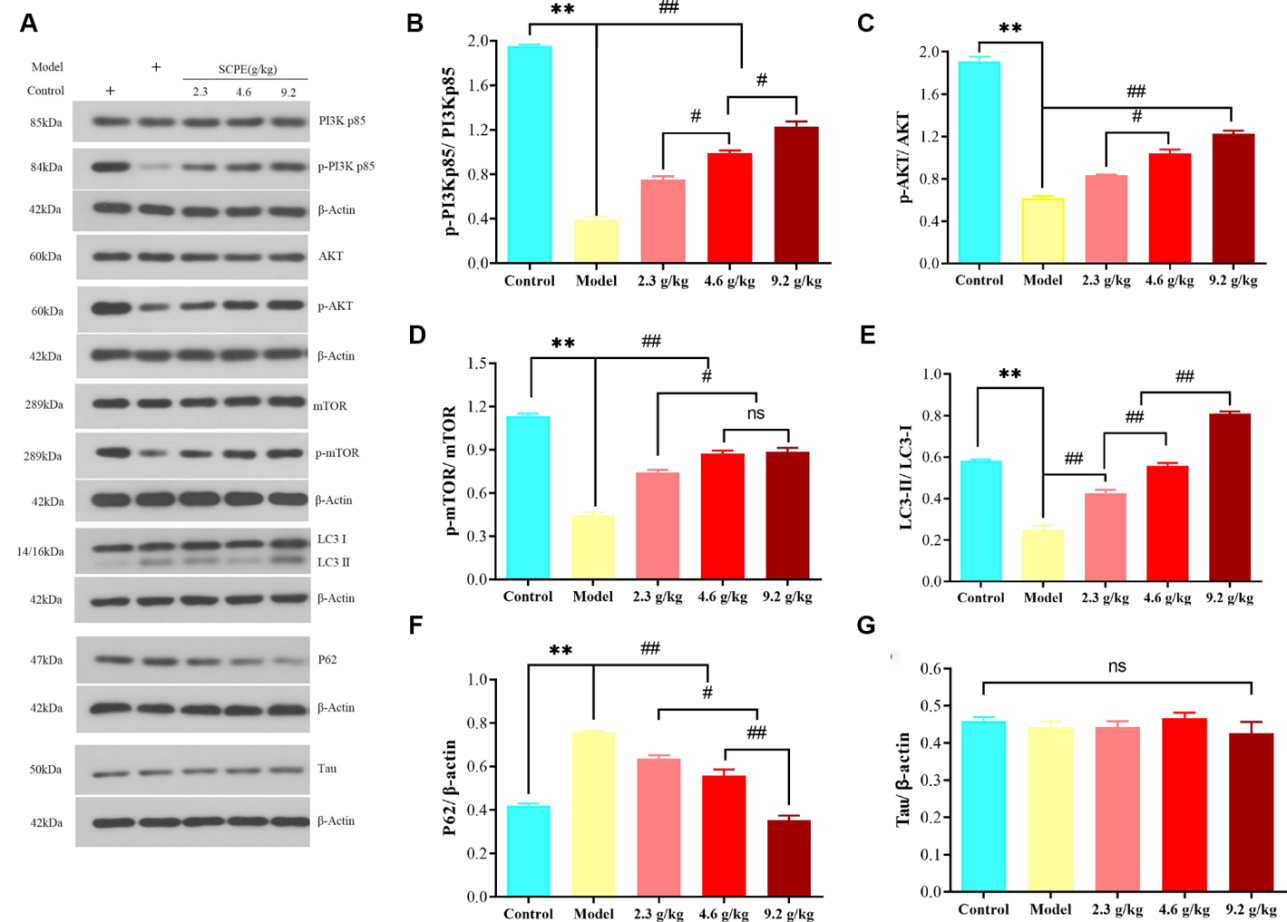
**Expression of proteins related to PI3K/AKT/mTOR signaling in the hippocampus.** (**A**) Western blot bands showing the levels of the p-PI3Kp85, PI3Kp85, p-AKT, AKT, p-mTOR, mTOR, LC3-II, LC3-I, P62, and Tau proteins in the hippocampus. (**B**–**G**) Quantification of the p-PI3Kp85/PI3Kp85, p-AKT/AKT, p-mTOR/mTOR, LC3-II/LC3-I P62, and Tau levels in the hippocampal tissue. The data are presented as means ± SD (*n* = 3). ^**^P < 0.01 compared with the model group; ^##^*P* < 0.01 and ^#^*P* < 0.01 compared with the 2.3 g/kg or 4.6 g/kg group. Abbreviation: ns: not significant.

The Western blot results for LC3-II/LC3-I, P62, p-PI3KP85/PI3Kp85, p-Akt/AKT, p-MTOR/mTOR, and Tau protein levels in the hippocampus of CI mice in each group are shown in Figure 12.

Compared with the control group, the ratios of p-PI3Kp85/PI3Kp85, p-AKT/AKT, p-mTOR/mTOR, and LC3-II/LC3-I in the hippocampus of the model group were significantly decreased (*P* < 0.01), and the expression level of the P62 protein was significantly increased (*P* < 0.01). Compared with the model group, the ratios of p-PI3Kp85/PI3Kp85, p-AKT/AKT, p-mTOR/mTOR, and LC3-II/LC3-I in the hippocampus of mice in the SCPE-treated groups were significantly increased (*P* < 0.01), but the expression level of the P62 protein was significantly decreased (*P* < 0.01). Compared with the 2.3 g/kg SCPE-treated group, the ratios of p-PI3Kp85/PI3Kp85, p-AKT/AKT, p-mTOR/mTOR, and LC3-II/LC3-I in the hippocampus of the 4.6 g/kg and 9.2 g/kg SCPE-treated groups were significantly increased (*P* < 0.01), and the expression level of the P62 protein was significantly decreased (*P* < 0.01). Compared with the 4.6 g/kg SCPE-treated group, the p-PI3KP85/PI3Kp85 and LC3-II/LC3-I ratios in the hippocampus of mice in the 9.2 g/kg SCPE-treated group were significantly increased (*P* < 0.01), and the expression level of the P62 protein was significantly decreased (*P* < 0.01). A significant change in the expression level of the Tau protein was not observed in the hippocampus of all the groups (*P* > 0.05).

## DISCUSSION

CI is a common disease caused by aging and other factors [32]. Years of clinical practice have suggested that a single targeted drug does not effectively treat CI, a complex disease with a strong biological network. Presently, a specific drug is unavailable for CI, and the main available treatment comprises symptomatic and supportive therapy. TCM has broad application prospects because of its extensive advantages, such as its diversity of targets and mechanisms. Previous studies have found that TCM not only targets the causes of CI but also effectively relieves a series of neurodegenerative diseases caused by aging [33, 34].

Kaixin San (KXS), SCPE and other Chinese herbal medicines [35] have been proven to improve hippocampal neurodegenerative changes and cognitive functions in patients with CI. SCPE contains various active components that directly target brain tissue to play a role in the treatment of CI, suggesting that it exerts a therapeutic effect that other drugs cannot. This effect has been confirmed in the systems pharmacology prediction part of this study. Our research team has long been committed to bioinformatics, basic mechanistic studies and evaluations of the clinical efficacy of TCM in preventing and treating CI. The mechanism of the treatment of CI by SCPE has been extensively discussed in previous studies, but TCM compounds often have multiple components and multiple targets, and certain limitations exist in the study of the effective components and basic properties of the mechanism of SCPE therapy for CI.

In recent years, systems pharmacology in the treatment of diseases has attracted extensive attention worldwide. Systems pharmacology and artificial intelligence technology represent effective methods to screen and predict drug targets. Based on these research methods, this study revealed the active components of SCPE involved in the treatment of CI and further revealed their potential mechanism of action. According to the GO analysis and most relevant signaling pathways identified by KEGG enrichment analysis, the PI3K/AKT/mTOR signaling pathway and pharmacodynamic Chinese medicine monomers in SCPE were selected to construct a PPI network and perform molecular docking verification.

Among the 23 selected monomers (Table 2), 2’-O-methylisoliquiritigenin (C5) has been proven by Deng, Lei et al. [36] to regulate neurotransmitters, including glutamic acid (Glu), γ-aminobutyric acid (GABA), 5-HT, epinephrine (E), dopamine (DA), and norepinephrine (NE), and exert sedative, hypnotic and anxiolytic effects. Olasehinde, Tosin A and colleagues [37] found that gentisin (C25) exerts antiamnestic effects by regulating cholinesterase, purinergase, and monoamine oxidase and reducing the redox imbalance in the brains of male rats treated with scopolamine. Additionally, it inhibits the proliferation of vascular smooth muscle cells [38]. However, the application of perlolyrine (C26), onjixanthone I (C22) and other monomers as treatments for CI has not been confirmed, but they inhibit phosphodiesterase type 5 (PDE5), have anti-inflammatory and antioxidant activities, and regulate immune function [39, 40]. These new discoveries of TCM monomers support further research.

Using the ADME/T model of systems pharmacology and Python programming language, we predicted the basic proprietary properties and molecular docking of 23 Chinese medicine monomers, suggesting that these monomers play a role in regulating the release of neurotransmitters through the PI3K/AKT/mTOR signaling pathway to improve synaptic plasticity and cognitive function. Some of these monomers are effective against CI. For example, Naidu, M et al. [41] found that euxanthone (C23) attenuates Aβ1–42-induced apoptosis and oxidative stress by enhancing autophagy; this neuroprotective effect on Aβ1–42 indicates a potential therapeutic role for euxanthone in AD. After treating PC12 cells with euxanthone butanone for 1 hour, the level of phosphorylated mitogen-activated protein (MAP) kinase was increased, indicating that euxanthone is a plant-derived compound that stimulates neurite growth. Zhou, Hui et al. [42] found that euxanthone exerts neuroprotective effects by upregulating NRF2 expression, which is related to the inhibition of sevoflurane-induced apoptosis and neuroinflammation. Additionally, Leong, Waikit et al. [43] found that patchoulene (C13) regulates certain bacteria that produce short-chain fatty acids (SCFAs) in C57BL/6J mice. Examples include anaerobes producing butyrate, *Buvibrio fibrinolyticus*, *Clostridium jejunum*, *Eugenia*, and *Lactobacillus* and their key SCFAS receptors GPR41, 43, and 109A. Patchoulene exerted an effect similar to prebiotics.

Among the 23 screened monomer components, *Acorus tatarinowii* contained the most active components (18). This finding is consistent with the theory of TCM that “*Acorus tatarinowii* is a holy medicine for improving memory”. Systems pharmacology can provide insights into the combination of modern medicine and TCM and explain the feasibility of TCM theory for guiding clinical drug use to a certain extent. At the same time, we found that 23 active ingredients were all from the herbs *Acorus tatarinowii* and *Polygala tenuifolia Willd* in SCPE, while the animal drug *Chinemys reevesii* (Gray) and mineral drug Fossilia Ossis Mastodi did not exert an effect on the PI3K/AKT/mTOR pathway. This result also supports the future development of Oriental medicine, namely, to optimize the composition of medicinal formulas and to clarify the active ingredients and mechanisms of compound medicines. Exploring new combinations of active ingredients combined with systems pharmacology is more beneficial for the development of Oriental medicine and the treatment of related diseases.

The critical role of the PI3K/AKT/mTOR pathway in autophagy has been reported in many previous experiments [44–46]. In the aging body, the inhibition of autophagy prevents the removal of excess protein in a timely manner, and excessive protein aggregation can lead to CI and other neurodegenerative diseases [47]. PI3K comprised the regulatory p85 subunit and catalytic p110 subunit. PI3Kp85 binds to a related adaptor protein or an activated receptor tyrosine kinase to activate the catalytic subunit. Thus, PI3Kp110 induces the activation of phosphatidylinositol 4,5-phosphate (PIP2) to produce phosphatidylinositol 3,4,5-triphosphate (PIP2), which induces AKT activation. Phosphorylated AKT continues to activate the downstream mammalian target of rapamycin (mTOR). mTOR, a serine/threonine protein kinase, is the intersection of multiple signaling pathways regulating autophagy. As a key factor in regulating autophagy, mTOR is the key intersection of multiple signaling pathways mediating autophagy. mTOR phosphorylation can significantly promote autophagy [48]. According to Razani, Elham et al. [49], the key pathological reactions in Alzheimer’s disease are regulated by the PI3K/AKT/mTOR pathway, particularly in regulating brain apoptosis and autophagy. Lee, Han-Kyu et al. [50] reported that mTORC2 overexpression plays a crucial role in the activation and regulation of neuronal autophagy in animal models of Alzheimer’s disease. At the same time, the promotion of PI3K/AKT/mTOR phosphorylation may be conducive to the clearance of Aβ and positively affects the prevention and treatment of CI. The results of Hu, Jiang-Yuan’s team [51] showed that stimulated PI3K positively regulated the level of 5-HT in the brain tissue of *Aplysia* and could produce long-term synaptic plasticity. Many studies have shown that the phosphorylation of PI3K/AKT/mTOR reduces the degree of brain tissue damage and cognitive decline, and insufficient phosphorylation leads to an inflammatory cascade reaction exacerbating necrosis and apoptosis [52]. These results are consistent with our predicted and experimentally validated trends. The advantage of our study is that it overcomes the limitations of the extensive previous PI3K/AKT/mTOR-based studies on cognitive disorders. In particular, our team studied cognitive disorders caused by aging and highlights the specific active components and mechanism of action of commonly used drugs in clinical practice. Additionally, autophagy mediated by the PI3K/AKT/mTOR pathway is closely related to LC3 and P62. LC3 is the mammalian homolog of yeast Atg8 protein, which forms type I Atg8 protein (LC3I) by catalytic cleavage of Atg4 cysteine protease. When autophagy occurs, LC3I will enzymatically hydrolyze some polypeptides under the action of Atg3 and Atg7 and turn into membrane-type LC3 (LC3II). Therefore, the higher is the ratio of LC3II/LC3I, the higher is the degree of autophagy; conversely, the lower the degree. The P62 protein is a selective substrate for autophagy and is degraded in the middle and late stages of autophagy. The level of P62 in cells is negatively correlated with the degree of autophagy [48]. Studies have shown [53] that the inhibition of neuron autophagy and inability to remove the excessive accumulation of Tau protein in the cell can also aggravate nerve fiber tangles to a certain extent.

In the present study, 4.6 g/kg and 9.2 g/kg of SCPE not only promoted the phosphorylation of the PI3K/AKT/mTOR signaling pathway and effectively increased the LC3II/LC3I ratio (*P* < 0.01) but also decreased the protein expression of P62 (*P* < 0.01). It also increased the levels of the neurotransmitters 5-HT and 5-HIAA in cerebrospinal fluid, alleviated brain tissue damage, promoted autophagy, and improved the cognitive function of SAMP8 mice. However, notably, in the senescence-induced CI model, abnormal expression of Tau protein in the hippocampus of SAMP8 mice was not observed, as previously reported [54]. The reason may be related to the specificity of senescence or a special induction factor. Additionally, combined with our previous study, SAMP8, as an animal model of CI, is characterized by amyloid deposition. These studies are valuable to reveal the pathogenesis and treatment of senescence-induced CI and reflect the advantages of the multitarget and multipathway mechanisms of TCM compounds. In the most recent study, Yao, Rubin [55] found that euxanthone inhibits traumatic spinal cord injury by inhibiting oxidative stress and regulating the p38 and PI3K/Akt signaling pathways. Euxanthone was also one of the 23 monomers predicted in this study (Table 2).

**Figure 13:**
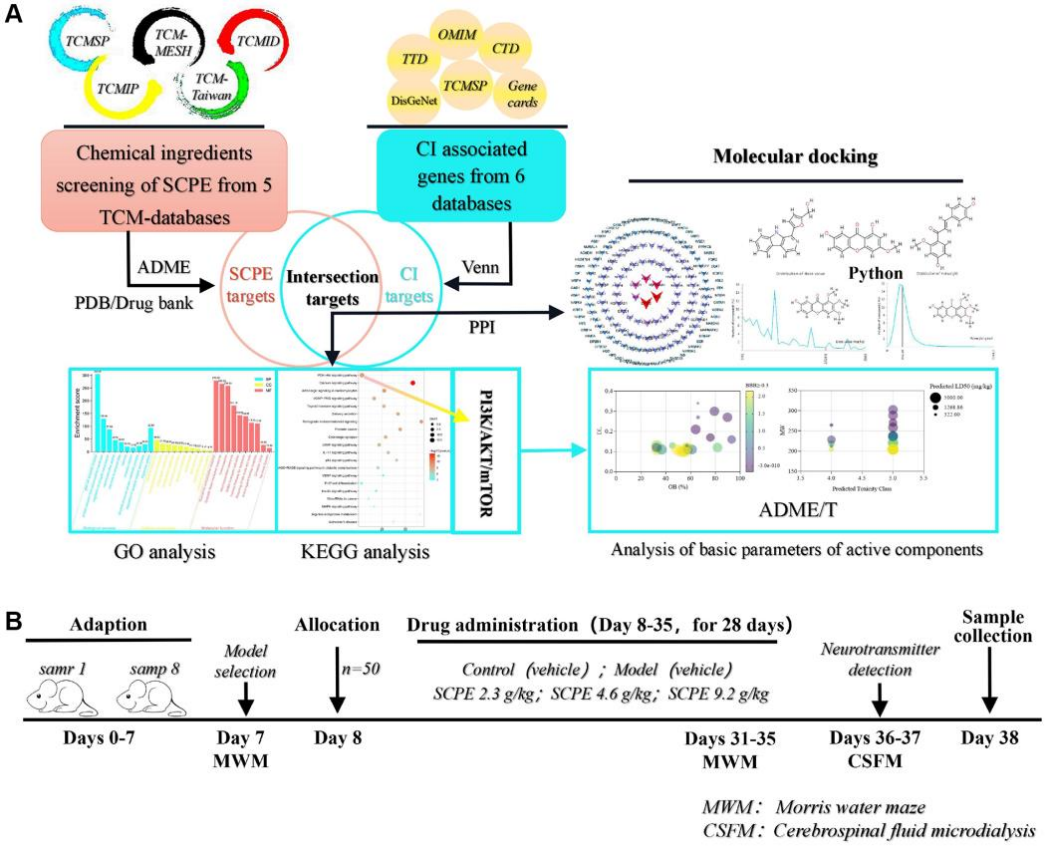
**Schematic diagram of the systems pharmacology and experimental protocol.** (**A**) Flowchart of the systems pharmacology approach for deciphering the therapeutic mechanisms of action of Sagacious Confucius’ Pillow Elixir (SCPE) in cognitive impairment (CI). (**B**) Flowchart of the experimental protocol.

SCPE not only exerts effects similar to those of PI3K/AKT/mTOR pathway activators but also exerts significant effects on improving brain tissue pathology, regulating the levels of monoamine neurotransmitters (5-HIAA and 5-HT) and improving cognitive function (Figure 13). Systems pharmacology is an effective approach to predict the active components and mechanism of action of SCPE in treating CI. This study provides a basis for using SCPE as a comprehensive treatment for CI.

## CONCLUSIONS

**Figure 14:**
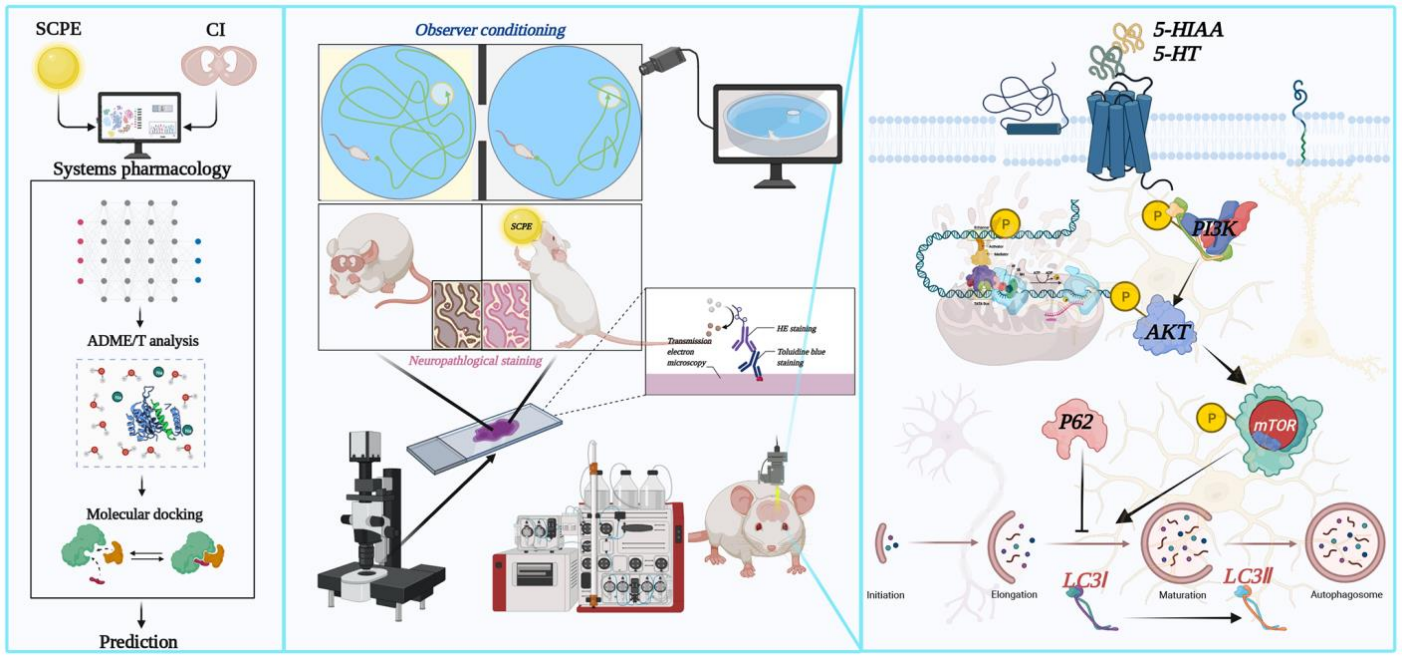
Overall structural diagram of the active components and molecular mechanisms of SCPE in the treatment of CI.

In summary, the experimental results described in this study suggest that SCPE improves senescence-induced cognitive dysfunction and protects against hippocampal injury. By performing systems pharmacology and drug toxicity risk assessments, 23 potentially active ingredients were identified in SCPE to treat CI. Additionally, the PI3K/AKT/mTOR pathway plays a crucial regulatory role in the mechanism of SCPE therapy for CI, and its overall structural diagram is shown in Figure 14.

Some limitations of this study require further study. These limitations include the need for in-depth studies of predicted monomers and combining the monomers of SCPE determined in this study with nanotechnology to create an advanced administration route for the targeted treatment of CI with TCM. Additionally, irrelevant drug studies using these formulations are needed. However, this study confirmed the feasibility of treating CI with classic SCPE prescriptions based on systems pharmacology. This study saves the time of exploration and cost of scientific research. Further study of CI monomers and related transcription factors with potential therapeutic effects is also possible, and the results are expected to provide a breakthrough in the treatment of CI with Oriental medicine.

## MATERIALS AND METHODS

### Data processing and network construction related to systems pharmacology methods

Active components of the SCPE were searched in the following 5 databases: traditional Chinese Medicine Systems Pharmacology (TCMSP) database [56], the Traditional Chinese Medicine Information Database (TCMID) [57], the Integrative Pharmacology based Research Platform of TCM (TCMIP) [58], TCM-Mesh [59], and TCM database @ Taiwan [60]. The following screening criteria were applied: oral bioavailability (OB) greater than or equal to 30%, drug-likeness (DL) greater than or equal to 0.1 and blood–brain barrier (BBB) permeability greater than or equal to −0.3. Using these criteria, the potential target genes of the active ingredients were identified, and the target gene database of these active ingredients was established using these genes. Cytoscape 3.7.1 was used for visual network analysis of active ingredients and action targets. The Database for Annotation, Visualization, and Integrated Discovery (DAVID) was used to analyze the enrichment of SCPE target genes, perform gene function analysis, and draw bubble maps using the ggplot2 installation R package. The GeneCards database [61], Comparative Toxicogenomics Database (CTD) [62], Therapeutic Target Database (TTD) [63], Database of Online Mendelian Inheritance in Man (OMIM) [64], DisGeNet database (https://www.disgenet.org/home/) [65] and TCMSP database [56] were searched to obtain information on differentially expressed genes in subjects with CI. A keyword search was performed with the terms “cognitive impairment”, “cognitive decline” and “cognitive dysfunction”, and duplicate genes were removed to obtain a nonredundant set of CI-related genes, which were combined with the action targets selected for SCPE to obtain the potential mechanistic targets of SCPE in the treatment of CI. The DAVID gene function analysis tool was used for gene enrichment analysis, and the Kyoto Encyclopedia of Genes and Genomes (KEGG) pathway bubble map and Gene Ontology (GO) enrichment map were drawn using R software. After the enrichment of SCPE target genes, key signaling pathways were selected, and an SCPE active component-target gene database was constructed to identify potential active components.

Based on 5 TCM-related databases and the ProTox database [66], we investigated the physical and chemical properties of monomers. The protein–protein interaction (PPI) networks of target compounds and differentially expressed genes were plotted using the STRING database [67]. The most significant genes were selected from the network for subsequent molecular docking analysis with the active ingredients. The receptor protein encoded by the selected gene was searched in the UniProt database [68]. We downloaded the 3D structures of proteins from the Structural Bioinformatics Protein Database (RCSB PDB) [69] research collaboration laboratory. The two-dimensional structures of molecular ligands were downloaded from the PubChem database (https://pubchem.ncbi.nlm.nih.gov/). From the basic information obtained for the compounds, we used software written in Python (Version 3.9.0) to determine the molecular weight and median lethal dose (LD50) value distribution of compounds and compared them with the molecular weight distribution and dose distribution information in the database. The specific settings applied in the program were as follows:

Id: The request ID used to retrieve this dataset, marking each individual request.
Name: This variable represents the compound name of the request if the type is entered using a name; otherwise, it is an empty string.
Smiles: This variable indicates the SMILE input if the canonical SMILE input type is used; otherwise, it is an empty string.
Acute_tox: If selected, acute toxicity prediction with median lethal dose (LD50), toxicity class and prediction accuracy data.
LD50: Predicted LD50 in mg/kg.
Tox_class: Predicted toxicity class (1–6).
Avg_similarity: Average similarity in % (float 0–100).
Pred_accuracy: Predicted accuracy in % (float 0–100).
Tox_targets: If selected, toxicity targets with similarity values.
Abbreviation: Short-form toxicity target (e.g., ANDR, AOFA, CRFR1..., as on the website).
Tox_target: Full name of the toxicity target.
Average_similarity_known_ligands: Similarity score in % (float 0–100).
Binding_probability_class: 0–3 (0 = no binding, 3 = probable binding), color-coded according to the scale presented on the website.
Average_pharmacophore_fit: Fit score in % (float 0–100).
Tox_models: If selected, contains data for all other computable models with name, prediction, and prediction confidence.
(Model name): Short name of a model.
Prediction: Boolean value for predicting activity or inactivity (1 = activity, 0 = inactivity).
Probability: A floating point value from 0 to 1 indicates confidence in the above results.

In addition to the compound prediction results, some information concerning the input compound is provided. In the diagram on the left, the distribution of LD50 values obtained from the calculation is shown. The mean value of the dataset is shown in red, and the predicted median lethal dose of the input compound is shown in black. The diagram on the right indicates the molecular weight (MW) distribution of compounds in our dataset. The mean MW is indicated as a red line, whereas the MW of the input compound is indicated as a black line.

The method for molecular docking is described below. (1) The SDF file for the compound was downloaded from PubChem, imported into Chembio3D for energy minimization, and then imported into MGL tools 1.5.6 and AutoDock v4.2.6/version for hydrogenation. The charge was calculated and distributed; the rotation key was set, and the file was saved in the pdbqt format. (2) The entries for the crucial target proteins were downloaded from the PDB (http://www.rcsb.org/) (the human protein was preferred, an original ligand with high structural similarity to the active component to be docked was preferred, and the one with the highest resolution was selected). (3) The protein was input into PyMOL (2.3.0) software to remove the original ligand and water molecules and then was imported into AutoDock Tools software (V1.5.6) for hydrogenation. The charge was calculated and distributed; the atomic type was specified, and the file was saved in the pdbqt format. (4) The original ligand of each protein was used as the center of the docking box. If no original ligand was available, the area near the reported key amino acid residues was used as the docking area for each protein [70–72]. The size of the grid box was set to 80 × 80 × 80 (the spacing of each grid was 0.375 Å), and the default settings were used for the other parameters. (5) The interaction mode was analyzed using PyMOL 2.3.0 and Ligplot v2.2.4.

A schematic diagram of the systems pharmacology protocol is shown in Figure 13A.

### SCPE preparation

SCPE was purchased from Beijing Tongrentang Co., Ltd., China (Beijing, China) and was dissolved in distilled water. An SCPE suspension with a concentration of 0.92 g/mL was prepared. A certain volume of the SCPE suspension was absorbed and diluted with the same volume of distilled water to achieve a concentration of 0.46 g/mL. Similarly, the SCPE suspension was diluted to a concentration of 0.23 g/mL.

### Main reagents and instruments

Sodium pentobarbital was purchased from Shanghai Shangyao Xinya Pharmaceutical Co., Ltd., (China). The BCA Protein Quantitation Kit, Protein Marker and Loading Buffer were purchased from Thermo Fisher Scientific (USA). We purchased 5-hydroxytryptamine (5-HT) and 5-hydroxyindole acetic acid (5-HIAA) hydrochloride from the China Institute for the Control of Pharmaceutical and Biological Products (China). All the antibodies used for Western blotting, including Tau, phosphatidylinositol 3-kinase (PI3K) p85, p-PI3Kp85, protein kinase B (AKT), p-AKT, mammalian target of rapamycin (mTOR), p-mTOR, LC3, P62 and β-actin antibodies, were provided by Beijing Boorsen Biotechnology Co., Ltd. (China).

### Experimental animals and drugs

Forty healthy 7-month-old specific-pathogen-free (SPF) male mice of the SAMP8 genotype and ten of the senescence-accelerated resistant mouse R1 (samr 1) genotype (weighing 22 ± 2 g each) were purchased from the Medical Animal Center of the First Affiliated Hospital of Tianjin University of Chinese Medicine (No. SCXK (Tianjin) 2015-0003). The mice were housed in the center for the safety evaluation of drugs at Heilongjiang University of Chinese Medicine in a specific pathogen-free environment (Animal Laboratory license No. SYXK (Hei) 2016004). SAMR1 mice were assigned to the control group (*n* = 10). SAMP8 mice were randomly divided into a model group (*n* = 10), a low-dose SCPE group (2.3 g/kg/d, *n* = 10), a medium-dose SCPE group (4.6 g/kg/d, *n* = 10) and a high-dose SCPE group (9.2 g/kg/d, *n* = 10). All the mice were provided free access to sterile feed and autoclaved water during the experiment. The experimental protocol was reviewed and approved by the Animal Care and Use Committee of Heilongjiang University of Chinese Medicine (No. 2018042301).

### Morris water maze test

The water maze comprises a circular plastic pool (100 cm in diameter; 50 cm deep) filled with water (32 cm deep; 22–24°C) and an escape platform (12 cm in diameter) placed at a fixed position in the first quadrant of the pool 2 cm below the surface of the water. The mice were trained to perform the water maze twice a day for 4 consecutive days. On the fifth day, the mice were tested without an escape platform. The time required to reach the platform was recorded (escape delay). The cutoff period for each test was set at 90 s. If the mice did not reach the platform within 90 seconds, they were gently guided onto the platform and remained there for 20 seconds. A Likean AK-025K, SONY IR COLOR CCD camera, SYSTEM: PAL700TVL (SONY Corporation, Tokyo, Japan) recorded their movements using artificial intelligence software (SuperMaze Version 3.3.0.0; Shanghai Xinrui Information Technology Co., Ltd., Shanghai, China). The latency period and total swimming distance in the target quadrant were measured in each acquisition test. The time spent in the target quadrant and number of crossings through the previous platform location (platform intersections) were measured during the probe test.

### Quantification of the neurotransmitter 5-HT and metabolite 5-HIAA

#### Embedding of the microdialysis cannula

The mice were anesthetized by an intraperitoneal injection of 1% pentobarbital sodium at a dose of 50 mg/kg. After the mice were anesthetized, the head was fixed within a mouse brain stereotaxic frame. A hole was drilled into the skull of each mouse to locate the CA1 region in the mouse hippocampus (2.1 mm behind the anterior fontanelle; 1.8 mm left), and a dummy cannula was fixed in place inside the hole at a depth of 2.7 mm. Screws were fixed beside the dummy cannula with nails, and then dental cement was used to cover the wound, securing the dummy cannula and screws to ensure the stability of the casing.

### Preparation of a high-performance liquid-phase electrochemical instrument

#### Mobile phase configuration

For the mobile phase, 13.8 g of Na_2_HPO4·H_2_O, 160 mg of sodium 1-octanesulfonate, 10 mg of EDTA·H_2_O and 149.2 mg of KCl were weighed and added to 100 mL of methanol; pure water was added to bring the volume to 1 L. The pH was adjusted to 3.0 with H_3_PO_4_, and then an organic membrane was used to pump and filter the solution.

#### Chromatographic conditions

A glass carbon working electrode (voltage 0.52 V), a silver reference electrode, and a SYKAM C18 analytical column were used (3 μm, 2.1 × 100 m). The flow rate of the mobile phase was set to 0.2 mL/min, and each injection was 20 μL. The thermostat was set to 40°C.

### Standard curve drawing

5-HT and 5-HIAA standards (1 mg) were accurately weighed. The stock solution was prepared with 0.1 mol/L of perchloric acid. Using a pipetting gun, the liquid was serially diluted to 500 pg/mL, 250 pg/mL, 125 pg/mL, 62.5 pg/mL, 31.25 pg/mL, 15.625 pg/mL, and 7.8125 pg/mL. The standard solution was assessed using high-performance liquid chromatography (HPLC), and the standard curves of the 2 analytes were drawn.

### Collection and measurement of cerebrospinal fluid

On the second day after cannulation, an inner cannula connected to a syringe and a cooled collecting vessel were inserted into the dummy cannula. Lactated Ringer’s solution was infused at a rate of 0.1 μL/min, and 30 μL was collected from each mouse into three tubes at intervals of 1 hour. After the second tube was full, the first tube was discarded. Immediately after collection, the fluid was subjected to HPLC. The output file of each sample was saved to calculate the peak areas of 5-HT and 5-HIAA, and the concentrations of 5-HT and 5-HIAA in the sample were calculated by fitting the data to the standard curve.

### Neuropathological assessment

The hippocampal tissues of mice were fixed with 4% neutral formaldehyde, dehydrated and embedded in paraffin. The specimen was cut into 6 μm thick pieces and stained with HE and toluidine blue. Pathologists observed the pathological changes in the hippocampus after HE and toluidine blue staining at 200, 400 and 800 magnifications under a microscope. Finally, HE staining was performed to observe pathological changes in the CA1 and CA3 regions of the hippocampus. Toluidine blue staining mainly indicates the shape and number of Nissl corpuscles in hippocampal neurons. The other part of the hippocampus was fixed with 2.5% glutaraldehyde/0.1 mol/L dimethyl arsenic acid sodium buffer (pH 7.4), dehydrated and embedded, and the ultrastructure of mitochondria, autophagosomes and other organelles in the hippocampus was observed using transmission electron microscopy.

### Western blotting

Total hippocampal protein was collected in lysis buffer, and the protein concentration was measured using a BCA kit according to the manufacturer’s instructions. The protein samples were separated by 10% SDS polyacrylamide gel electrophoresis (SDS–PAGE) and transferred to a PVDF membrane, which was blocked with 10% bovine serum albumin for 2 h and then incubated with antibodies against the target protein and reference protein GAPDH for 4 nights. The membrane was rinsed with TBST 3 times for 10 min each and incubated with the secondary antibody at room temperature for 2 h. The membrane was rinsed with TBST 3 times for 10 min each and then incubated with an enhanced chemiluminescence (ECL) photoluminescence solution before imaging was performed, and the protein levels were analyzed using ImageJ software. The levels of the target proteins (LC3-II, LC3-I, P-PI3KP85, PI3Kp85, P-Akt, AKT, P-MTOR, mTOR, P62, Tau) were analyzed.

### Experimental schedule

The mice were allowed to acclimate for 7 days (days 0–7), and the animal model was screened on the 7th day. According to our previous screening criteria, animals with an escape latency >80 seconds in the water maze experiment were selected for the CI model and used in subsequent experiments. The animals were allocated to groups on the 8th day and maintained until the 35th day. A water maze experiment was conducted during the last 5 days of management (days 31–35) to assess changes in the animals’ cognitive performance. *In vivo* cerebrospinal fluid microdialysis was performed to detect the 5-HIAA and 5-HT levels within 2 days after the water maze experiment (days 36–37). Sampling was performed on day 38 for subsequent pathological and molecular biological studies. An overall schematic diagram of the animal experimental protocol is shown in Figure 13B.

### Statistical analysis

SPSS 22.0 and GraphPad Prism 9.2.0 software were used for statistical analysis and data visualization by statisticians in this study. The data were presented as means ± standard deviation. All the data were collected and analyzed in a blinded manner. The target latency, total swimming distance, time spent in the target quadrant, and number of platform crossings in the Morris water maze test were analyzed using one-way ANOVA, two-way ANOVA, or two-way repeated-measures ANOVA combined with the Bonferroni post hoc test. The Student-Newman–Keuls test was also used for multiple comparisons. Student’s *T* test (comparison between two groups) was used for data with a normal distribution and homogeneity of variance. Nondifferential tests were used when the variance was not uniform. *P* < 0.05 was considered statistically significant, and *P* < 0.01 was considered statistically highly significant.

## Supplementary Materials

Supplementary Figures
