# Supplementary material for: Active components and molecular mechanisms of Sagacious Confucius’ Pillow Elixir to treat cognitive impairment based on systems pharmacology
Source: Aging (Albany NY). 2023 Jul 30;15(14):7278–307. doi: 10.18632/aging.204912 (PMC10415554; doi:10.18632/aging.204912)
Supplement: Supplementary Figures [file aging-15-204912-s001.pdf]

SUPPLEMENTARY FIGURES

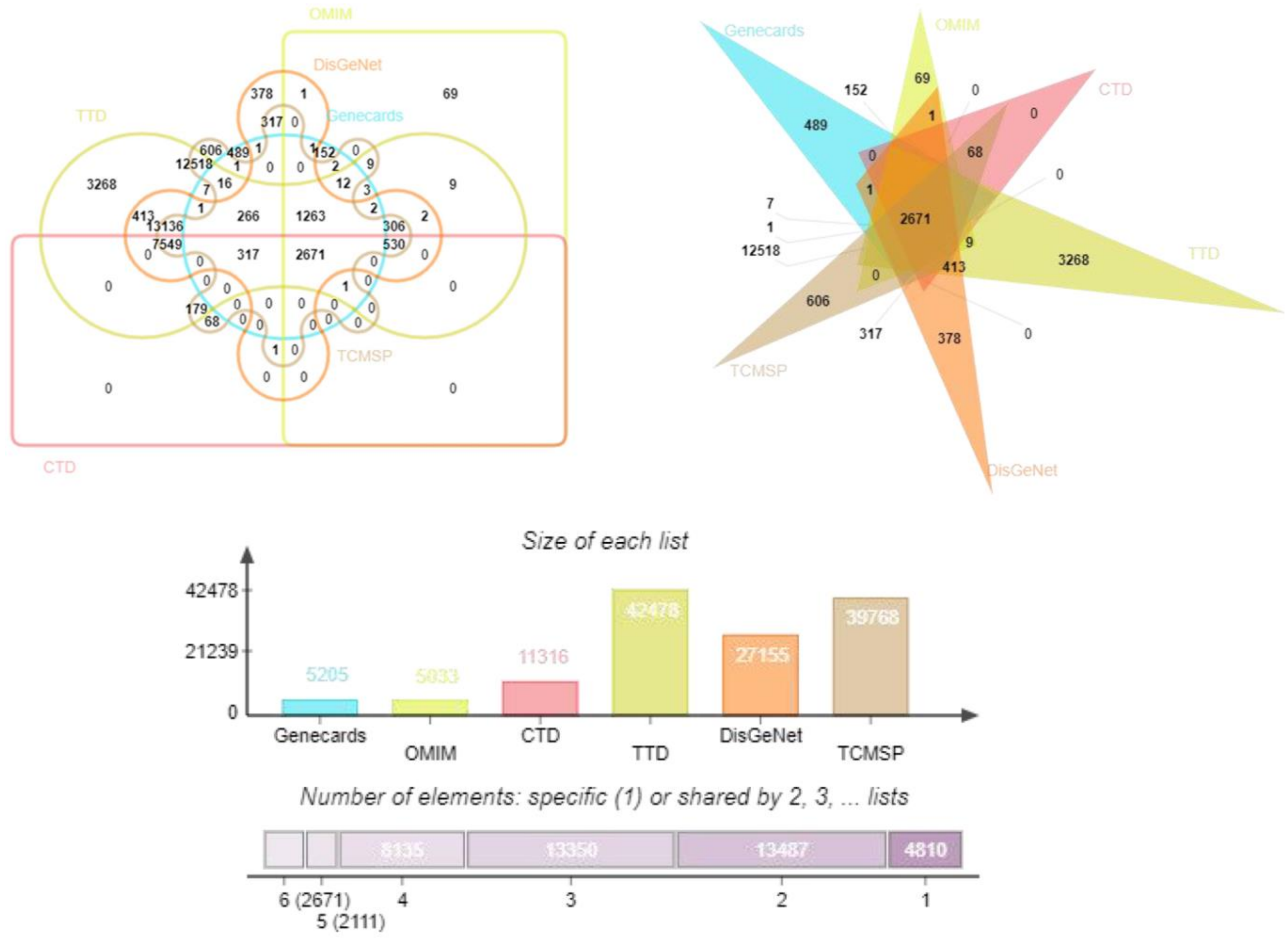

Supplementary Figure 1. Predictive results of active components of SCPE and targets involved in the treatment of CI based on systems pharmacology.

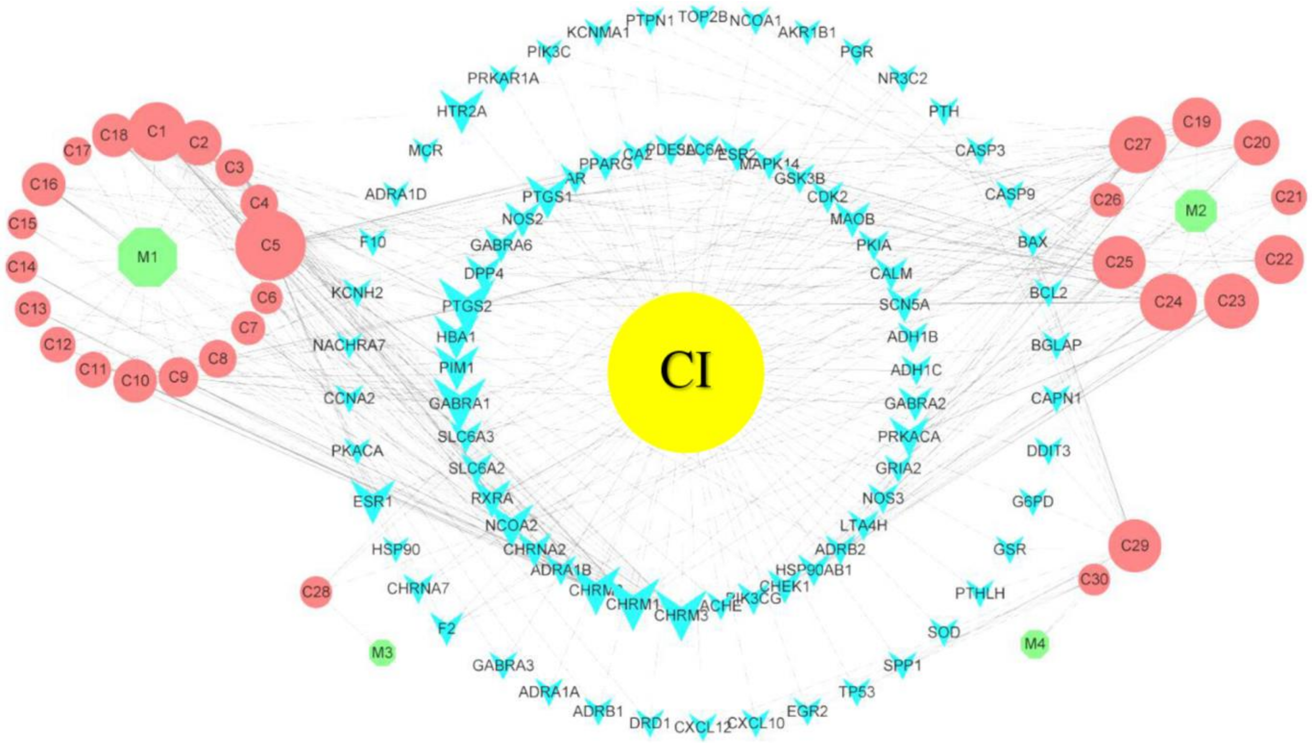

Supplementary Figure 2. Global compound-target-disease network of the candidate ingredients of SCPE used to treat CI. The size of each icon reflects its importance in the overall network. The yellow node represents the disease, the green nodes represent the herbs, the red nodes represent the compound, and the blue nodes represent the targets. Abbreviation: CI: cognitive impairment.

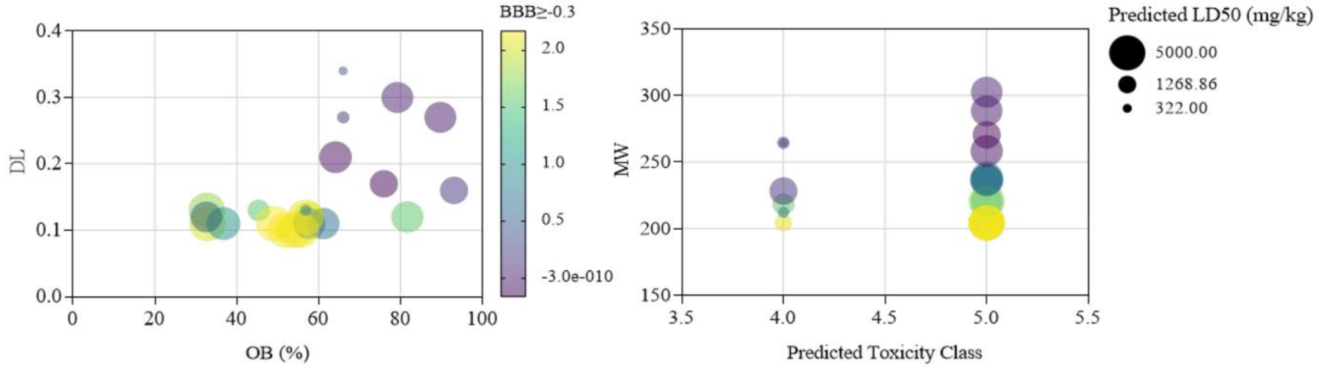

Supplementary Figure 3. Distribution of the pharmacological and toxicological parameters of the 23 active components of SCPE.

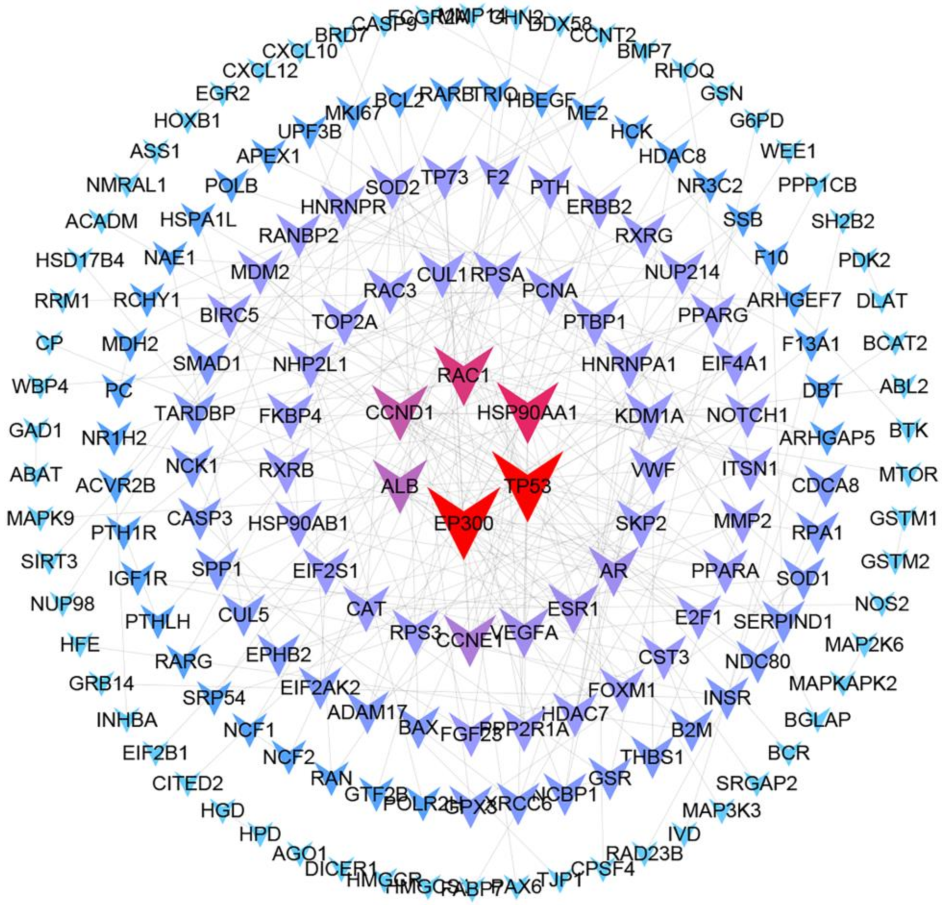

Supplementary Figure 4. SCPE alters the CI protein interaction network diagram.
